# Identification of biological pathway and process regulators using sparse partial least squares and triple-gene mutual interaction

**DOI:** 10.1038/s41598-021-92610-4

**Published:** 2021-06-23

**Authors:** Junyan Hong, Chathura Gunasekara, Cheng He, Sanzhen Liu, Jianqin Huang, Hairong Wei

**Affiliations:** 1 School of Forestry and Biotechnology, Zhejiang Agricultural and Forestry University, Linan, Zhejiang 311300 People’s Republic of China; 2 State Key Laboratory of Subtropical Silviculture, Zhejiang Agricultural and Forestry University, Linan, Zhejiang 311300 People’s Republic of China; 3 Department of Pediatrics, Baylor College of Medicine, USDA/ARS Children’s Nutrition Research Center, Houston, TX 77030 USA; 4 Department of Plant Pathology, Kansas State University, Manhattan, KS 66506 USA; 5 College of Forest Resources and Environmental Science, Michigan Technological University, Houghton, MI 49931 USA

**Keywords:** Computational biology and bioinformatics, Plant sciences

## Abstract

Identification of biological process- and pathway-specific regulators is essential for advancing our understanding of regulation and formation of various phenotypic and complex traits. In this study, we applied two methods, triple-gene mutual interaction (TGMI) and Sparse Partial Least Squares (SPLS), to identify the regulators of multiple metabolic pathways in *Arabidopsis thaliana* and *Populus trichocarpa* using high-throughput gene expression data. We analyzed four pathways: (1) lignin biosynthesis pathway in *A. thaliana* and *P. trichocarpa*; (2) flavanones, flavonol and anthocyannin biosynthesis in *A. thaliana*; (3) light reaction pathway and Calvin cycle in *A. thaliana*. (4) light reaction pathway alone in *A. thaliana*. The efficiencies of two methods were evaluated by examining the positive known regulators captured, the receiver operating characteristic (ROC) curves and the area under ROC curves (AUROC). Our results showed that TGMI is in general more efficient than SPLS in identifying true pathway regulators and ranks them to the top of candidate regulatory gene lists, but the two methods are to some degree complementary because they could identify some different pathway regulators. This study identified many regulators that potentially regulate the above pathways in plants and are valuable for genetic engineering of these pathways.

## Introduction

A multitude of biological processes and metabolic pathways are present in a plant species, and our understanding of their underlying regulatory mechanisms remains limited. For example, *Arabidopsis thaliana* has 549 annotated metabolic pathways and a few thousand biological processes, but the regulators for almost all of these pathways except a few of them have not yet been identified^1,2^. With the advent of the high-throughput sequencing technology and the mounting gene expression data being deposited into public databases, there is a great need for well-evaluated computational tools that can be utilized to predict pathway regulators using high-throughput gene expression datasets.

Identification of biological process and pathway regulators is very important to understand the regulation of various physiological and biochemical characteristics, and phenotypical and complex traits as well. This can be accomplished through constructing gene regulatory networks operating above biological processes and pathways^3,4^, where the connectivity of each transcription factor (TFs) and pathway genes, and network complexity and structures can convey the information based on which potential candidate regulators can be identified for experimental validation^5^. However, there are some inherent challenges for gene association or network construction due to the linear multicollinearity and high dimensionality in high-throughput gene expression data sets. A multitude of gene variables with respect to a small number of samples can easily result in linear multicollinearity. Both high dimensionality and multicollinearity impede us from accurately associating regulatory genes-target genes or constructing gene regulatory network via mathematical modeling.

To circumvent such obstacles, we need to develop novel algorithms and assess their performance and efficacy. SPLS regression methodology was designed to deal with the high dimension and multicollinearity property of gene expression data^6^. The main principle of SPLS is to impose sparsity within the context of partial least squares and thereby accomplish dimension reduction and variable selection simultaneously. SPLS regression performs well even when the sample size is much smaller than the number of variables. TGMI is specially developed for identifying the pathway regulators by evaluating all combined triple gene blocks using a novel mutual interaction measure (MIM) calculated with mutual information and conditional mutual information^7^. MIM represents the regulatory strength exerted by a transcription factor (TF) on two combined pathway genes in a triple gene block, and it can facilitate the recognition process of true causal relationships between TFs and pathway genes. Comparison TGMI with SPLS allows us to learn their performance, efficacy, commonality and specification, and utilize them for different purposes and applications.

In this study, we compared the efficiency of TGMI and SPLS in identifying regulatory factors of several metabolic pathways in two species, which include lignin biosynthesis pathway in *Arabidopsis thaliana*, a unified flavanone, flavonol and anthocyannin biosynthesis pathway in *A. thaliana*, lignin biosynthesis pathway in *Populus trichocarpa*, a unified pathway of light reaction and Calvin cycle in *A. thaliana*, and finally the light reaction pathway in *A. thaliana.* These unified pathways contain two to three closely linked pathways. We selected these pathways because their regulators have been mostly or partially identified. We set out to test the viability of combining multiple pathways in identifying their regulators. At the same time, it also provides clues for researchers to explore new regulatory mechanisms of these complex pathways. The results show that TGMI and SPLS are instrumental for identifying true biological pathway regulators. However, TGMI algorithm has an overall higher efficacy than SPLS algorithm and may identify more positive known regulators than SPLS.

## Results

### Lignin, flavanone, flavonol, and anthocyanin biosynthesis as well as photosynthesis pathways in Arabidopsis thaliana and Populus trichocarpa

The pathway genes we analyzed in this study were mainly acquired from Plant Metabolic Network (PMN) (https://plantcyc.org/). A complete gene list of flavanone, flavonol and anthocyannin biosynthesis pathway is provided in Table S1. The lignin pathway genes are listed in Table S2. The photosynthesis pathway genes, which include light reaction pathway genes and Calvin cycle pathway genes are provided in Table S3. To aid the understanding of the metabolic pathways we analyzed, we also plotted a diagram for lignin, flavanone, flavonol and anthocyanin pathways (Figure S1), and a diagram for photosynthesis pathway (Figure S2), which enable us to visualize the catalytic function of each enzymatic protein. We did not provide poplar lignin pathway diagram because it is largely similar to the one of *A. thaliana*’s. The poplar lignin pathway genes were extracted from Phytozome’s annotation file based on their counterparts’ annotation in *A. thaliana* (Table S4).

### Identification of regulators controlling the unified pathway of flavanone, flavonol and anthocyannin biosynthesis in A. thaliana

**Figure 1:**
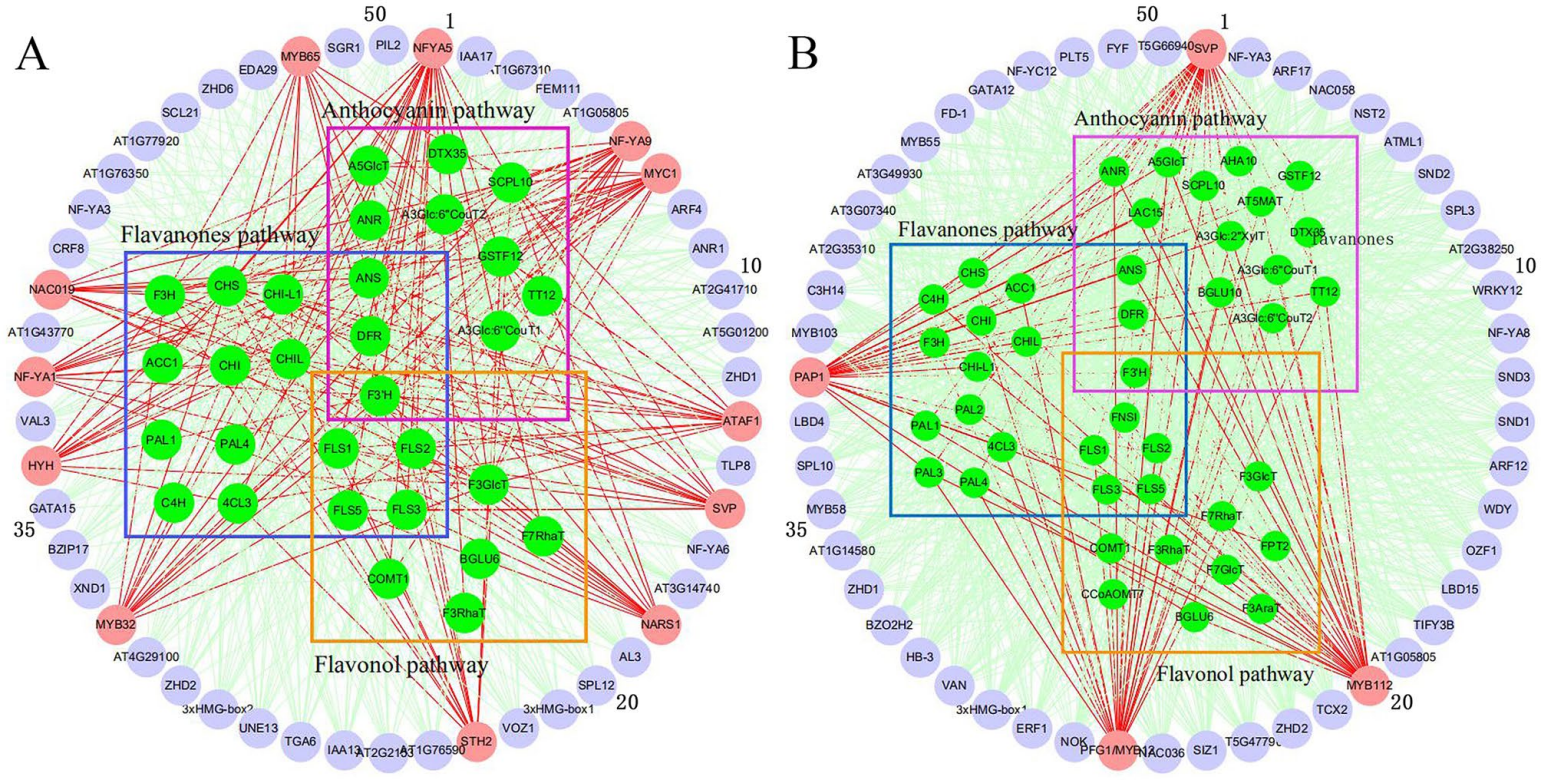
Regulatory network generated by triple-gene mutual interaction (TGMI) (**A**) and sparse partial least squares (SPLS) (**B**) for the *Arabidopsis thaliana* flavanone, flavonol and anthocyannin biosynthesis pathway using the microarray data generated from hypocotyledonous stem tissues. The green nodes represent pathway genes. All other nodes are the top 50 transcription factors that have the highest connectivity to pathway genes regardless of their colors. The light coral nodes represent positive known TFs of flavanone, flavonol and anthocyannin biosynthesis pathway while the red edges are to show the connections of a true positive known pathway regulator with pathway genes.

The efficacy of TGMI algorithm and SPLS algorithm in identifying pathway regulators was scrutinized using Data Set 1 from *A. thaliana* stems. The expression data of the genes involved in flavanones pathway, flavonol pathway and anthocyanin pathway (Table S1) and all transcription factors were extracted from the data. The resulting regulator lists (Table S5), and gene regulatory networks yielded from TGMI and SPLS methods are shown in Fig. 1A,B, respectively. TGMI identified 12 positive TFs while SPLS identified 4 positive TFs. Among the 12 positive TFs identified by TGMI method, NFYA5 enhances drought stress by regulating the accumulation of purple flavonoid pigment anthocyanin^8^. NF-YA1 and NF-YA9 overexpression in *A*. *thaliana* plants causes the brown pigment precipitation in the seed coat^9^. NARS1 regulates the accumulation of anthocyanins in epidermal cells. Flavonoid biosynthesis is regulated by MBW (MYB-bHLH-WDR) protein complexes^10^. MYC1 is a known component of MYB-bHLH-WD Repeat (MBW) transcriptional complex that controls flavonoid^11^. By DNA affinity purification sequencing (DAP-seq), ATAF1 is a predicted regulator that controls flavonoid synthesis pathway genes, including *C4H* and *CHS*^12^. SVP affects the accumulation of flavonol and anthocyanin in drought-stressed *Arabidopsis* plants^13^. HY5 induces biosynthesis of flavonoids by regulating the expression of *DFR* at low temperature^14^. STH2 has been reported to enhance anthocyanin accumulation by interacting with HY5^15^. MYB32 regulates the accumulation of flavonoids by interfering with the transcriptional activity of the MBW complex^16^. NAC019 negatively regulates the biosynthesis of anthocyanins^17^. MYB65 participates in regulating the accumulation of isoflavone^18^. Of the TFs identified by SPLS, MYB112 promotes the formation of anthocyanins^19^. PFG1 (PRODUCTION OF FLAVONOL GLYCOSIDES1)/MYB12 and PAP1 (PRODUCTION OF ANTHOCYANIN PIGMENT1)/MYB75 promote the accumulation of flavonoids under oxidative and drought stress^20^.

### Identification of lignin biosynthesis pathway regulators in A. thaliana

**Figure 2:**
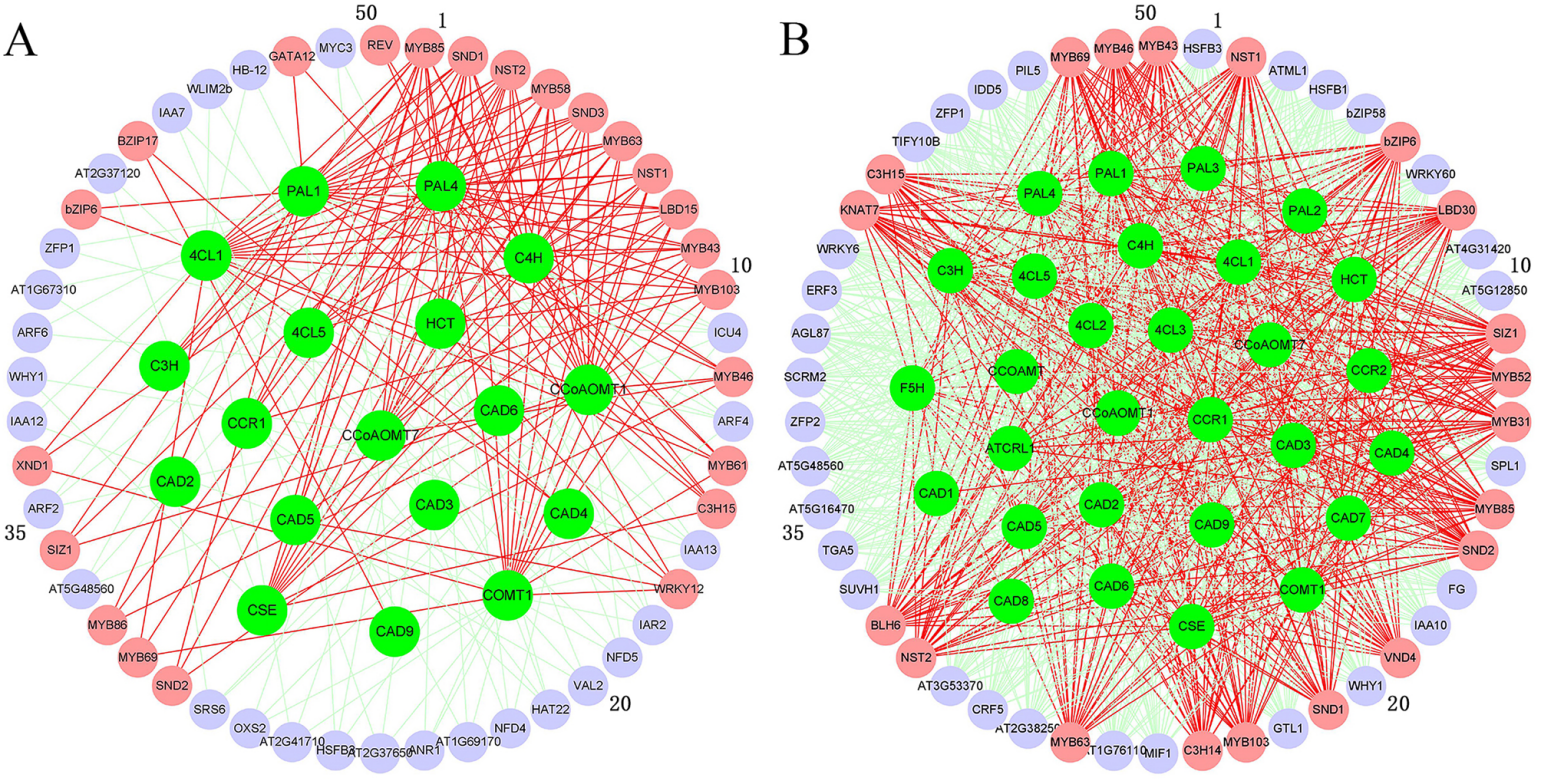
Regulatory network generated by triple-gene mutual interaction (TGMI) (**A**) and sparse partial least squares (SPLS) (**B**) for *Arabidopsis thaliana* lignin biosynthesis pathway using the microarray data generated from hypocotyledonous stem tissues. The green nodes represent pathway genes. All other nodes are the top 50 transcription factors that have the highest connectivity to pathway genes regardless of their colors. The light coral nodes represent positive known TFs of lignin biosynthesis pathway while the red edges are to show the connections of a true positive known pathway regulator with pathway genes.

TGMI and SPLS methods were tested for their accuracies in identifying lignin biosynthesis pathway regulators using the *A. thaliana* microarray compendium data set (Data Set I) produced from stem tissues where wood formation was intensified by short-day treatment. The expression data of the genes involved in lignin pathway (Table S2) and all transcription factors were extracted from the data set. The two lists containing the top 50 TFs that control the lignin biosynthesis pathway inferred by TGMI and by SPLS (Table S6), and gene regulatory networks constructed by the two methods are shown in Fig. 2A,B, respectively. The positive lignin biosynthesis pathway regulators, which are indicated as a lignin pathway regulator by literature, are shown in coral color. TGMI identified 23 known lignin pathway regulators while SPLS identified 20 positive TFs. It is perceptible that more positive known TFs identified by TGMI were ranked at the top of regulatory candidate gene list.

In *A. thaliana*, the five transcription factors, *SND1*, *NST1*, *NST2*, *VND6*, and *VND7,* belong to the secondary cell wall NAC (SWN) group, and serve as transcription switches to activate secondary cell wall biosynthesis^21,22^. Five additional VND genes, *VND1-5*, have recently been proven to have similar functions to SWN genes^23,24^. Among the above genes, SND1 is a master transcriptional activator of secondary wall synthesis. The transcription factors regulated by it include *SND2*, *SND3*, *MYB20*, *MYB42*, *MYB43*, *MYB46*, *MYB52*, *MYB54*, *MYB58*, *MYB61*, *MYB63*, *MYB69*, *MYB83, MYB85*, *MYB86*, *MYB103*, *KNAT7*, *C3H14* and *C3H15*^25,26^. TGMI algorithm identified 15 SWN genes and their downstream regulatory factors (*SND1*, *NST1*, *NST2*, *SND2*, *SND3*, *MYB43*, *MYB46*, *MYB58*, *MYB61*, *MYB63*, *MYB69*, *MYB85*, *MYB86*, *MYB103* and *C3H15*). SPLS algorithm also identified 15 TFs (*SND1*, *NST1*, *NST2*, *SND2*, *VND4*, *MYB43*, *MYB46*, *MYB52*, *MYB63*, *MYB69*, *MYB85*, *MYB103*, *KNAT7*, *C3H14* and *C3H15*). In addition to these TFs, TGMI also identified 8 other known lignin pathway regulators (*LBD15*, *WRKY12*, *SIZ1*, *XND1*, *GATA12*, *REV*, *bZIP6*, *BZIP17*) while SPLS identified 5 other known regulators (*bZIP6*, *LBD30*, *SIZ1*, *MYB31*, *BLH6*). GATA12 and LBD15 are upstream transcription factors that regulate the transcription of *VND7*^27,28^. WRKY12 binds to the promoter of *NST2*, thereby negatively regulating the biosynthesis of lignin^29^. AtSIZ1, a small ubiquitin-related modifier (SUMO) E3 ligase, regulates the formation of secondary cell walls in *A. thaliana* by mediating the SUMOylation of transcription factor *LBD30*^30^. XYLEM NAC DOMAIN 1 (XND1) inhibits xylem differentiation and secondary wall synthesis^31^. BLH6 and KNAT7 together form the KNAT7-BLH6 complex, which inhibits the formation of secondary cell walls, and *REV* is the direct target of this complex^32^. MYB31 has been shown to be involved in regulation of lignin synthesis genes multiple species^33^. bZIP6 and bZIP17 also regulate secondary cell wall synthesis^34,35^.

### Prediction of lignin biosynthesis pathway regulators in Populus trichocarpa

**Figure 3:**
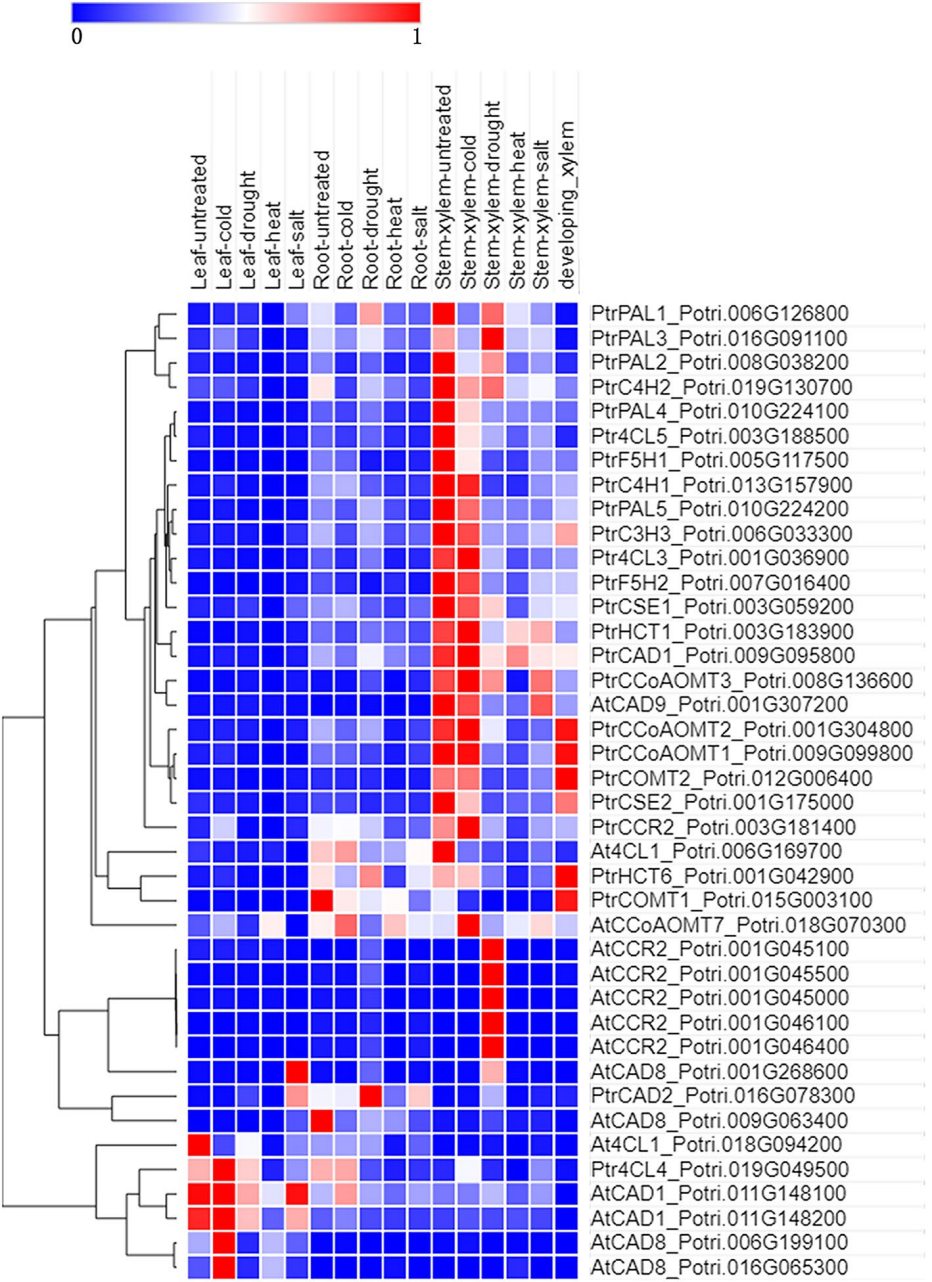
Hierarchical clustering of the expression profiles of lignin biosynthesis pathway genes in different tissues of *Populus trichocarpa*.

According to the verified lignin biosynthesis genes and the annotation information of the genes collected from Phytozome (https://phytozome.jgi.doe.gov/)^36,37^ (Table S4). We identified 40 *P. trichocarpa* lignin pathway genes whose expression patterns in Data Set 3 as represented by a heatmap are shown in Fig. 3. Most of these genes are highly expressed in xylem. Among them, five homologous genes of *AtCCR2 (AT1G80820)*, *Potri.001G045000*, *Potri.001G045100*, *Potri.001G045500*, *Potri.001G046100*, *Potri.001G046400,* were highly expressed only in drought-treated xylem, and may be specifically involved in poplar drought stress response in tree xylem.

**Figure 4:**
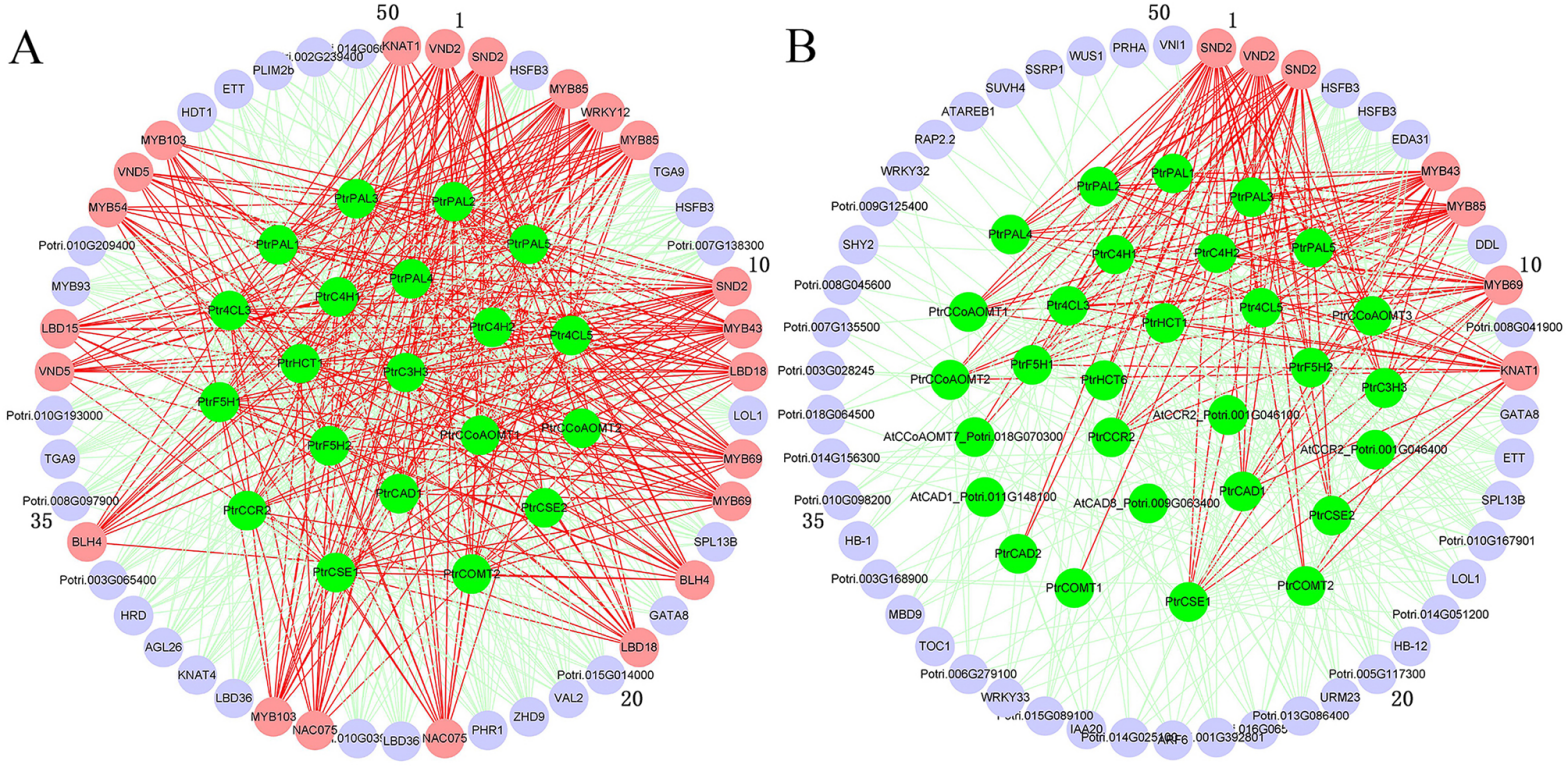
Regulatory network generated by triple-gene mutual interaction (TGMI) (**A**) and sparse partial least squares (SPLS) (**B**) for the *Populus trichocarpa* lignin biosynthesis pathway using the RNA-seq data generated from developing xylem tissues. The green nodes represent pathway genes. All other nodes are the top 50 transcription factors that have the highest connectivity to pathway genes regardless of their colors. The light coral nodes represent positive known TFs of lignin biosynthesis while the red edges are to show the connections of a true positive known pathway regulator with pathway genes.

**Figure 5:**
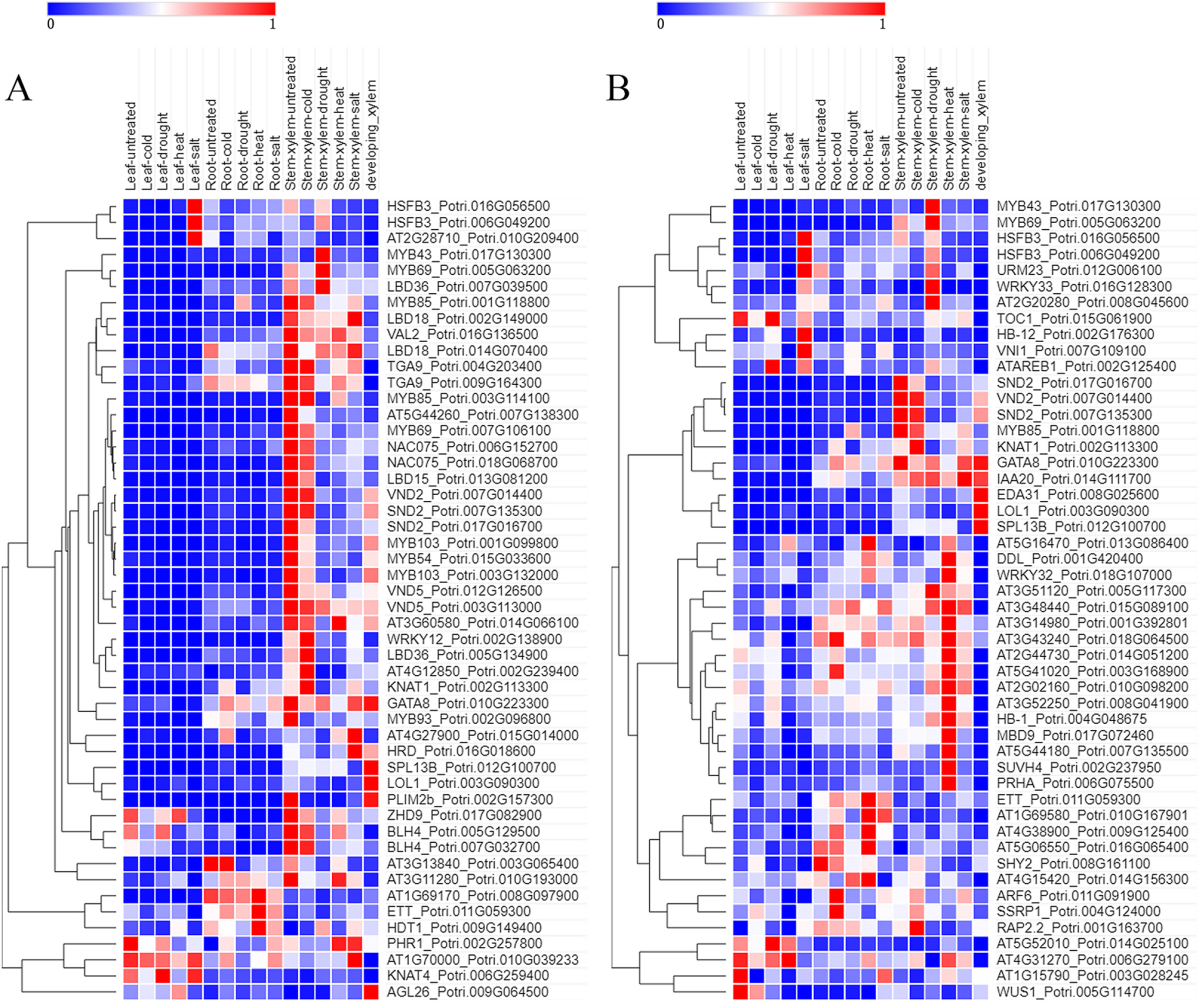
Hierarchical clustering of the expression profiles of lignin biosynthesis regulators identified with triple-gene mutual interaction (TGMI) (**A**) and sparse partial least squares (SPLS) (**B**) in different *Poplar* tissues. Only the top 50 transcription factors that have the highest connectivity to pathway genes are shown.

We used genes from lignin biosynthesis pathway to study which regulatory genes can potentially control wood formation in *P. trichocarpa*. The compendium data set we used comprises of 134 RNA-seq data sets from developing xylem. The input expression data for all TFs and 36 pathway genes were extracted from Data Set 3. The resulting regulator lists identified TGMI and SPLS (Table S7), and gene regulatory networks constructed by the two methods are shown in Fig. 4A,B, respectively. Among the top 50 TFs regulators, which interfere with the pathway genes with highest frequencies, in the lists identified by TGMI and SPLS, 22 and 7 are known lignin pathway regulators, respectively. In addition, 7 and 5 lignin biosynthesis pathway regulators are the counterparts of those identified by TGMI and SPLS from *A. thaliana* data (Data Set 1) (Fig. 2A,B), indicating that the methods can identify conserved TFs across two species. Moreover, some other positive TFs were also identified: Two homologous genes of *AtNAC075 (Potri.006G152700, Potri.018G068700)*, *AtLBD18 (Potri.002G149000, Potri.014G070400)*, *AtBLH4 (Potri.005G129500, Potri.007G032700)*, one homologous gene of *AtKNAT1(Potri.002G113300)*. The overexpression of NAC075, an upstream regulator of *VND7*, induces secondary cell wall deposition^24^. LBD18 participates in the positive feedback loop of *VND7* and regulates tracheary elements differentiation-related genes^38^. KNAT1 regulates vascular cambium development and xylem differentiation^39^. The gene regulatory networks of the lignin pathway constructed by two methods are shown in Fig. 4A,B, respectively. We also analyzed the expression profiles of TFs identified by TGMI and SPLS in the three tissues of leaves, roots and xylem using Data Set 3, and the results are shown in Fig. 5A,B, respectively. Most of the regulatory genes identified by TGMI also exhibited a higher expression level in xylem than root tissue, and the expression pattern in root tissue was greater than that in leaf tissue, especially positive TFs. However, the regulatory genes identified by SPLS did not exhibit such expression patterns. This supports that the aptitude for recognition of xylem-specific regulators by TGMI surpassed SPLS.

### Identification of regulators controlling the unified photosynthesis pathway of light reaction and Calvin cycle in A. thaliana

**Figure 6:**
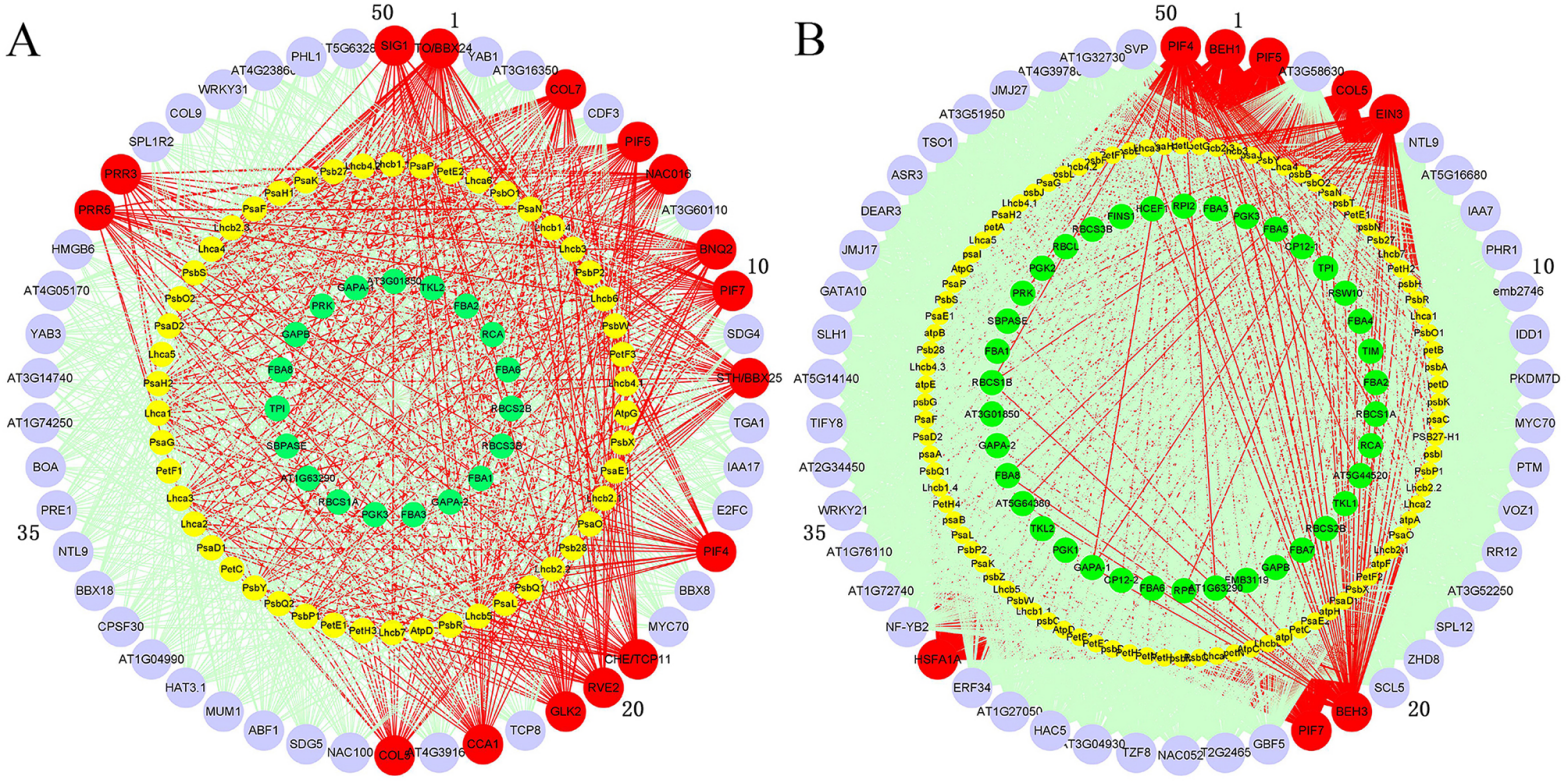
Gene regulatory network produced by triple-gene mutual interaction (TGMI) (**A**) and sparse partial least squares (SPLS) (**B**) algorithms for *Arabidopsis thaliana* light reaction and Calvin cycle pathway using the RNA-seq data generated from leaf tissues. The yellow nodes represent light reaction pathway genes. The green nodes represent Calvin cycle pathway genes. All other nodes are the top 50 transcription factors that have the highest connectivity to pathway genes regardless of their colors. The red nodes represent known positive regulatory genes that affect light reaction and Calvin cycle pathways while the red edges are to show the connections of a true positive known pathway regulator with pathway genes.

We used genes from the unified pathway of light reaction and Calvin cycle to study which regulatory genes can potentially control photosynthesis. The compendium data set, Data Set 3, comprises of 686 RNA-seq timeseries data sets from *A. thaliana* leaves under Mock, JA or SA treatments. Expression data for all TFs and 130 pathway genes were extracted from the above compendium data set and used for analyses. The resulting regulator lists identified and ranked by TGMI and SPLS (Table S8), and gene regulatory networks constructed by the two methods are shown in Fig. 6A,B. Among the top 50 TFs that were ranked by their interference frequencies on pathway genes in descending order, 16 positive known TFs showed up in the list identified by TGMI and 8 positive known TFs in the list identified by SPLS. The gene regulatory networks of the pathway constructed by TGMI and SPLS are shown in Fig. 8A,B, respectively.

The circadian clock regulates a large number of biological processes in plants, including light harvesting, electron transport in photosynthesis and ATP concentration in chloroplasts^40^. In *A. thaliana*, more than 30% of nuclear coding genes and 70% of chloroplast genes are regulated by circadian rhythms^41,42^. A group of genes, Lhca, Lhcb and Rubisco, which are involved in photosynthesis, have been shown to be regulated by circadian rhythms^43,44^. Three *A. thaliana* genes that play a critical role in the regulation of circadian rhythm are *CIRCADIAN CLOCK ASSOCIATED 1 (CCA1)*, *LATE ELONGATED HYPOCOTYL (LHY)*, and *TIMING OF CAB EXPRESSION 1 (TOC1)*^45,46^. They form the central oscillator of the circadian clock and a double negative feedback loop. In the morning, CCA1 and LHY bind to the promoter region of *TOC1*, reducing its mRNA abundance; at night, TOC1 inhibits the transcription of *CCA1* and *LHY*^47,48^. *TOC1* is a member of the PRR (PSEUDO-RESPONSE REGULATOR) family that includes *PRR9*, *PRR7*, *PRR5*, *PRR3*, and *PRR1/TOC1*. In addition to TOC1, PRR9, PRR7, PRR5, PRR3, and CCA1 HIGH EXPEDITION (CHE)/TCP11 also inhibit *CCA1* at different times of the day^49,50^.

The circadian clock controls the transcriptional expression of chloroplast genes by regulating a class of nuclear-encoded sigma factors, thereby affecting the production of photosynthetic apparatus and photosynthetic efficiency^51,52^. There are 6 sigma factors, *SIG1-6* in *A. thaliana*. SIG1 and SIG5 function in adjusting the photosynthetic apparatus during photosynthesis, and SIG2 and SIG6 are necessary for chloroplast development. It has been proved that that SIG1, SIG2 and SIG3 regulate *psaA*, *PIF4*, and *psbN*, respectively^53,54^, and SIG5 regulates *psaA*, *psaB*, *psbB*, *psbH*, *petB*, *petD*, *psbD*, and *psbC*^51,55^. Phytochrome interacting factors (PIFs) are a class of bHLH transcription factors that regulate the response of plants to light. PIF1, PIF3, PIF4 and PIF5 have been shown to affect the expression of photosynthesis-related genes, including genes encoding LHCA, LHCB, and PsaD proteins. PIF7 is also involved in the regulation of circadian rhythms^56,57^.

Among the aforementioned known regulators of photosynthesis, TGMI algorithm identified 8 regulators, namely, *CCA1*, *PRR3*, *PRR5*, *CHE/TCP11*, *SIG1*, *PIF4*, *PIF5* and *PIF7*. SPLS algorithm identified 3 regulators, *PIF4*, *PIF5* and *PIF7*. In addition to these regulators, TGMI also identified 8 other known photosynthesis pathway positive regulators, namely, *STO/BBX24*, *STH/BBX25*, *COL7*, *COL5*, *NAC016*, *BNQ2*, *REV2* and *GLK2*) while SPLS identified 5 other known positive TFs, namely, *BEH1*, *BEH3*, *COL5*, *EIN3* and *HSFA1A*. BBX24 and BBX25 physically interact with photosynthesis regulator *HY5* to inhibit its transcriptional activation activity^58,59^. CIR1/RVE2 have been shown to affect the transcription of Lhcb family genes by regulating *CCA1*, *LHY* and *TOC1*^60^. The two genes of the GOLDEN TWO-LIKE (GLK) family, GLK1 and GLK2, directly regulate the expression of a series of photosynthetic genes, including the genes encoding the PSI-LHCI complex and PSII-LHCII complex^61,62^. EIN3 directly interacts with PIF3, inhibiting the expression of most light-harvesting complex (LIGHT COMPLEX, LHC) genes^63^. Photoreceptors, phyA and phyB are required for stabilization of the COL7 protein, and COL7 is a critical factor linking light perception to changes in auxin level in *A. thaliana*^64^. *BNQ2* and *COL5* are regulated by PIF4 and their proteins participate in the integration of optical signals and GA signals^65^. NAC016 can promote the degradation of chlorophyll by directly increasing the transcription of *STAYGREEN1 (SGR1)*, and may affect the detoxification of chlorophyll by SGR-CCE-LHCII complex^66^. HSFA1, a master regulator of transcriptional regulation under heat stress, regulates photosynthesis by inducing the expression of downstream transcription factors^67^. *BEH1* and *BEH3* are homologous genes of *BZR1*, genetic analysis indicates that the BZR1-PIF4 interaction controls a core transcription network by integrating brassinosteroids and light response^68^. It is worth mentioning that there are 4 B-box family genes (*BBX8*, *BBX18*, *BBX24*, *BBX25*) in the top 50 TFs in TGMI recognition results. The genes of this family are involved in many plant processes regulated by light, so the role of *BBX8* and *BBX18* in photosynthesis is worth noting. It is noticeable that the positive known regulators identified by TGMI congregated at the top of the list (Fig. 6A).

**Figure 7:**
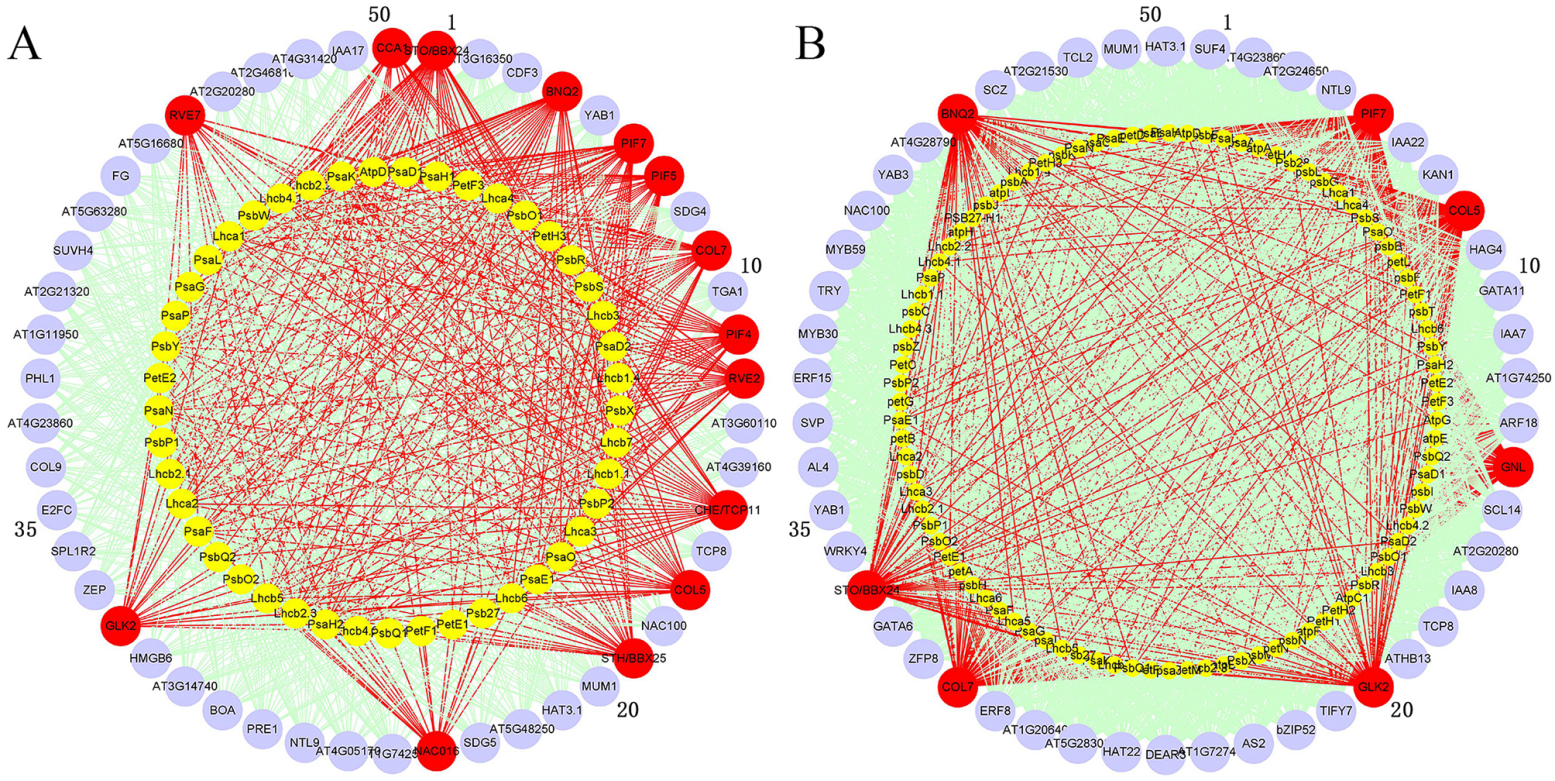
Gene regulatory networks produced by triple-gene mutual interaction (TGMI) (**A**) and sparse partial least squares (SPLS) (**B**) for *Arabidopsis thaliana* light reaction pathway using the RNA-seq data generated from leaf tissues. The yellow nodes represent light reaction pathway genes. All other nodes are the top 50 transcription factors that have the highest connectivity to pathway genes regardless of their colors. The red nodes represent known positive regulatory genes that affect light reaction pathway while the red edges are to show the connections of a true positive known pathway regulator with pathway genes.

In addition to comparing the power of the two methods on photosynthesis pathway regulators, we also analyzed the light reaction pathway separately to compare whether the combination of two metabolically contiguous pathways can achieve higher accuracy in identifying true pathway regulators than use of a single pathway and the results are shown in Fig. 7A,B, and Table S9. Among the top 50 TFs identified by the two methods, TGMI identified 14 positive TFs while SPLS identified 7 positive TFs. The gene regulatory networks are shown in Fig. 7A,B. Compared with the analysis of the combined pathway, these two methods have a slight decreased efficiency in identifying positive TFs. Comparing the two output lists of identified positive TFs by TGMI, light reaction pathway and unified pathway have largely the same as regulators. Three regulators, *PRR3*, *PRR5*, and *SIG1* are absent, but *RVE7* was identified^69^. The list of positive TFs identified by SPLS is quite different in these two analyses. There are seven positive TFs in SPLS’s list of light reaction pathway regulators, which are *PIF7*, *COL5*, *GNL*, *GLK2*, *COL7*, *STO/BBX24* and *BNQ2*. Among these seven genes, only *PIF7* and *COL5* appear in SPLS's list of the unified pathway. Based on the above analysis, we believe that the combination of two or more pathways may increase the accuracy of identifying the pathway regulators, and enable us to have a more comprehensive understanding of regulation of multiple linked pathways.

### The performance and sensitivity of TGMI and with SPLS

To compare the performance of TGMI and SPLS, we summarized the results we obtained from the above analyses. The result is shown in Table 1. TGMI could identify more positive known TFs than SPLS given the same input files. In five independent pathway analyses, TGMI identified at least 12 and at most 23 positive TFs in the top 50 TFs that were identified to regulate each pathway. Compared with the more stable recognition efficiency of TGMI, the number of positive TFs identified by SPLS varied greatly with different pathways, with a minimum of 4 and a maximum of 20 positive known TFs. On the other hand, the two methods have identified some different regulators in most cases, which indicates that the two they have their uniqueness and can be used complementarily for identifying pathway regulators. In addition, the running time of SPLS algorithm was several fold of that of TGMI algorithm (Table 1).

**Table 1:** Summary of identifying pathway regulators using triple-gene mutual interaction (TGMI) and sparse partial least squares (SPLS) in the top 50 transcription factors that have the highest connectivity to pathway genes.

Species	Tissues	Pathway	Number of positive TFs			Runtime TGMI/SPLS
			TGMI	SPLS	In common	
*Arabidopsis*	Stem	Flavanone, flavonol, anthocyannin	12	4	1	2.24/16.3 h
*Arabidopsis*	Stem	Lignin	23	20	13	0.64/20.09 h
*Populus*	Xylem	Lignin	22	7	7	0.85/7.14 h
*Arabidopsis*	Leaf	Photosynthesis (Light reaction + Calvin cycle)	16	8	4	12.91/70.24 h
*Arabidopsis*	Leaf	Light reaction	14	7	6	–/–

*These computing times were measured on Unix Intel(R) Xeon(R) CPU X5460 @ 3.16 GHz, with 2 physical ID, 4 cpu cores, 8 processors.

**Figure 8:**
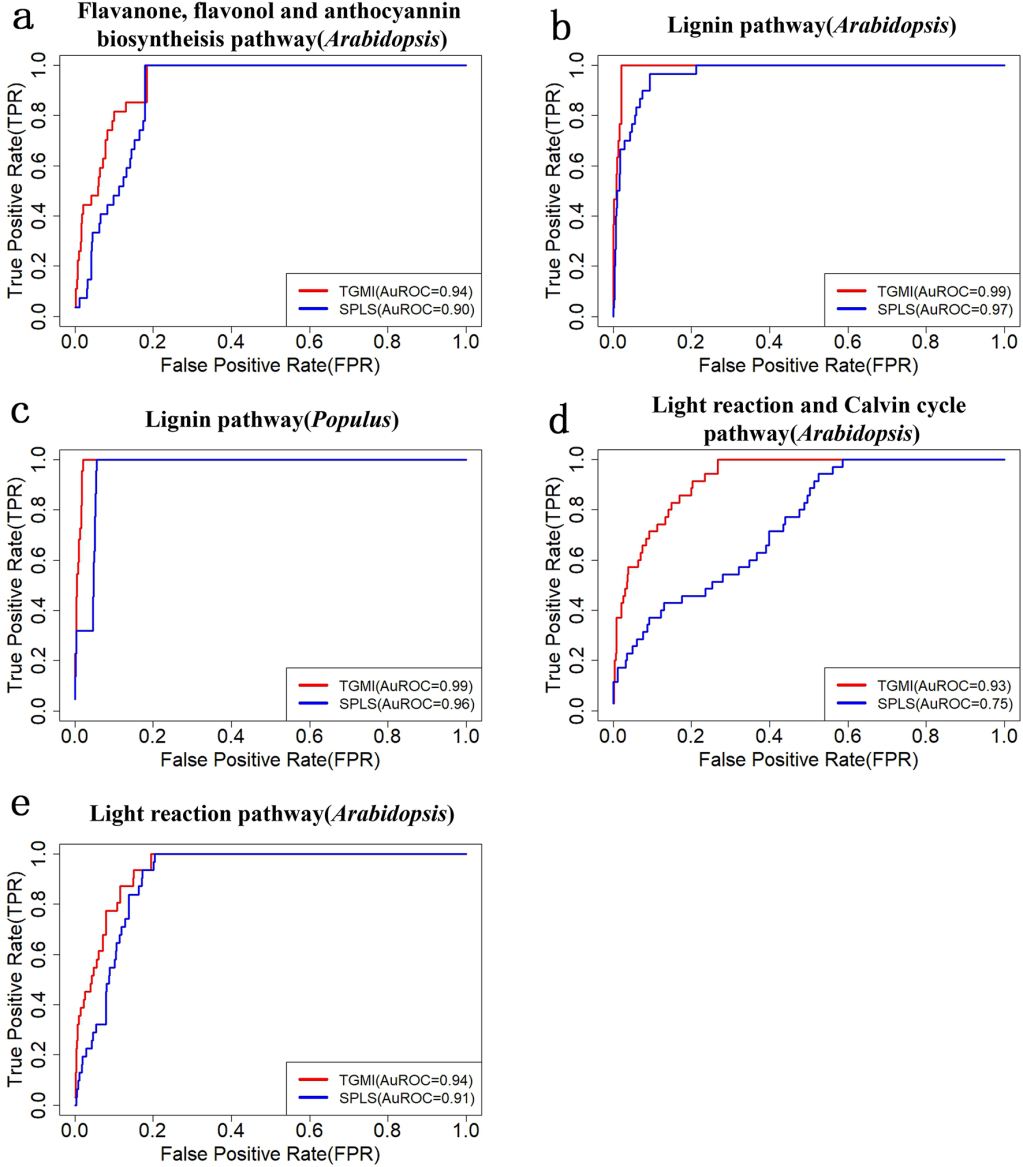
The receiver operating characteristics (ROCs) of TGMI and SPLS algorithms in recognition of pathway regulators. The ROC curves that closely follow the TPR axis, and then the top FPR axis represent the higher sensitivity in identifying positive regulators. The area under the ROC curve (AUROC) was also calculated to show which methods had high higher sensitivity versus 1-specificity in correctly ranking the candidate regulatory pathway genes^70,71^.

Finally, the receiver operating characteristic (ROC) curves of all pathways we analyzed are shown in Fig. 8. The area under ROC (AUROC) values of TGMI in all pathways were greater than 0.93. Except for the light reaction and Calvin cycle pathway (0.75), the AUROC values of SPLS for all pathways were also greater than 0.90.

## Discussion

Our studies on TGMI and SPLS support that TGMI has higher efficacy in recognition of the potential pathway regulators than SPLS. As summarized in Table 1, TGMI recognized more regulators than SPLS in all pathways. The ROC curve also manifested the higher sensitivity of TGMI in ranking the positive regulatory genes at the top of the candidate regulatory gene lists than SPLS given the same specificity. The comparison of AUROC values also confirmed this; TGMI had a larger AUROC value for all pathways, indicating that it persistently ranks the true positive regulators at the top of candidate list. This is an important advantage because biologists can only evaluate small number of genes at the top of candidate regulatory gene lists.

To examine what kinds of pathway regulators the two method can identify in different species, we compared the inferred lignin biosynthesis pathway regulators between poplar and *A. thaliana*. For *A. thaliana*, we identified a lignin biosynthesis regulatory network with several SWN genes (*SND1*, *NST1*, *NST2*, *SND2*, *SND3* and *VND4*) known as the main switches. In *Populus.* we only identified 4 SWN orthologs, which include *Potri.007G135300* (*SND2*), *Potri.017G016700* (*SND2*); *Potri.007G014400* (*VND2*); *Potri.012G126500* (*VND5*). We also identified two homologous genes of *NAC075 (Potri.006G152700, Potri.018G068700)*, two homologous genes of *LBD18 (Potri.002G149000, Potri.014G070400)*. *NAC075* and *LBD18* are the upstream regulators of *VND7*, which is known to regulate a handful of downstream lignocellulosic biosynthesis pathway regulators. These findings indicate that pathway regulators in perennial woody plants and annual herbs may be different. It is worth to mention that *NAC075* and *LBD18* were identified by TGMI rather than SPLS, suggesting TGMI is probably more sensitive to recognize pathway regulators.

We investigated if a combination of multiple pathways for analysis may be beneficial more than analyzing each pathway separately. We combined light reaction pathway and photosynthesis pathway as unified pathway for analysis. We found that combining two linked and coordinated pathways can improve the efficiency in identifying their regulators to a certain extent. The application of TGMI and SPLS algorithms for identifying light reaction pathway regulators in *A. thaliana* resulted in 14 and 7 positive regulators, respectively. The application of TGMI and SPLS algorithms for identifying the combined pathway regulators in *Arabidopsis* resulted in 16 and 8 positive regulators, respectively. Although we do not know if a combination will certainly lead to identification of more true positive regulators in all cases, it is worth undertaking. As evidenced, we identified CCA1 and SIG1 master regulators when the two photosynthesis and light reaction pathways were combined.

## Conclusion

The application of two methods to five metabolic pathways in *A. thaliana* and *P. trichocarpa* demonstrated that TGMI in general performs more efficiently than SPLS. TGMI tends to rank more true positive TFs at the top of candidate regulatory gene lists. This is an advantage given the fact that biological experimental validation can only be applied to the top candidate genes. In addition, TGMI appears to has more stable recognition efficiency in identifying true positive pathway regulators of multiple pathways in both species. In the two candidate regulatory gene lists of the same pathway yielded by TGMI and SPLS, there were always some different regulatory genes, indicating that the two methods are also complementary to each other to some degree. Moreover, both methods can be used to identify regulators for a unified pathway of several closely linked pathways, which may possibly increase the potential for identifying regulators that control both one or multiple pathways. Finally, we showed that TGMI is capable of identifying more tissue-specific pathway regulators than SPLS. In conclusion, both methods are instrumental for identifying pathway regulators from high-throughput data though TGMI is more efficient than SPLS for identifying positive known and/or tissue-specific regulators.

## Materials and methods

### Data Set 1: Arabidopsis microarray data set (128 chips) from stem

The *Arabidopsis* stem compendium dataset contains 128 Affymetrix microarrays pooled from six experiments (accession identifiers: GSE607, GSE6153, GSE18985, GSE2000, GSE24781, and GSE5633 in NCBI Gene Expression Omnibus (GEO) (http://www.ncbi.nlm.nih.gov/geo). These datasets were originally obtained from hypocotyledonous stems under short-day conditions known to induce secondary wood formation. The original CEL files were downloaded from GEO and preprocessed using the affy package in Bioconductor (https://www.bioconductor.org.) and then normalized with the robust multi-array analysis (RMA) algorithm in affy package. This compendium data set was also used in our previous studies^7^. The annotation information of all genes was acquired from the *Arabidopsis* Information Resource website (TAIR) (https://www.arabidopsis.org/), and the list of all transcription factors was acquired from the PlantTFDB website (http://planttfdb.cbi.pku.edu.cn/).

### Data Set 2: Arabidopsis RNA-seq data set from leaf (686 libraries)

The *Arabidopsis* leaf compendium dataset contains 686 RNA-seq data sets downloaded from NCBI BioProject database (https://www.ncbi.nlm.nih.gov/bioproject), with an accession number of PRJNA224133. These datasets were originally obtained from the 6th leaves from the apical buds in time series with three treatments: mock, salicylic acid (**SA**) or jasmonic acid (**JA**). This project contains 172 experiments; each has 4 samples except for the one that only has two samples. In total, there are 686 samples. Raw reads were trimmed to remove adaptors and low‐quality base pairs via Trimmomatic (v3.3). Clean reads were aligned to the *Arabidopsis thaliana* TAIR10 genome with STAR, followed by the generation of normalized FPKM (fragments per kb of transcript per million reads) using Cufflinks software (v2.1.1)^72^. The annotation of all genes and transcription factors was obtained from TAIR and PlantTFDB as described in Data Set 1.

### Data Set 3: Populus trichocarpa RNA-seq data sets from developing xylem

The *Populus trichocarpa* compendium data set comprising of 134 developing xylem samples was downloaded from NCBI database: BioProject (https://www.ncbi.nlm.nih.gov/bioproject) with an accession number of PRJNA300564. Raw reads were aligned to the *P. trichocarpa* v4.0 genome with TopHat (v2.1.1), followed by generation of raw counts using Bedtools^73^. Finally, the raw counts were normalized with TMM (Trimmed Mean of M values) using edgeR package^74^. The annotation information of all genes including transcription factors was acquired from the Phytozome website (https://phytozome.jgi.doe.gov/) and the list of all transcription factors was acquired from the PlantTFDB website (http://planttfdb.cbi.pku.edu.cn/).

### Data Set 4: Populus trichocarpa RNA-seq data sets from leaves, roots and stems (controls and stresses) for heatmap and two-way cluster analysis

RNA-seq data sets of different tissues of *Populus trichocarpa* (Nisqually-1) were acquired by sequencing the 81 RNA-seq libraries made from the total RNA isolated from three tissues, mature vascular leaves, stem xylem and roots, which were sampled from plants subjected to cold, heat, drought and high salinity treatments. The sequencing reads were downloaded from NCBI database: BioProject (https://www.ncbi.nlm.nih.gov/bioproject) with an accession number of PRJEB19784. Raw reads were aligned to the *P. trichocarpa* v4.0 genome with TopHat (v2.1.1), followed by the generation of raw counts using Bedtools^73^. Finally, the raw counts were normalized with TMM (Trimmed Mean of M values) contained in edgeR package^74^. The annotation information of the transcription factors was acquired from Phytozome website (https://phytozome.jgi.doe.gov/).

### Principle of TGMI

Triple-gene mutual interaction (TGMI)^7^ calculates the mutual information and conditional mutual information among a triple-gene block (Two pathway genes and one TF) using high-throughput gene expression data, and then evaluates if there are causal relationships among the triple genes. The significance of causal relationships was determined by bootstrapping. This algorithm was developed based on two biological phenomena: one is that genes within the same biological pathway/process or closely related biological pathways/processes are often more tightly co-expressed^75^; the other one is that genes with same or similar expression patterns are often under the regulation of the same molecular mechanism^76,77^. In addition, previous studies have showed that triple gene block is better than pairwise gene block for capturing causal relationships^7,78,79^. TGMI does not have tuning parameters and was implemented in R^7^. It can be can be download from: http://sys.bio.mtu.edu/sample_output/TGMI/.

### Principle of SPLS

Sparse partial least squares (SPLS)^6^ is a very effective method for achieving independent variable reduction given a set of dependent variables in high-dimensional data sets. It has a very wide range of applications for variable selection in high-dimensional genomic data with multicollinearity. PLS regression for either a univariate or multivariate response provides consistent estimators only under restricted conditions, and the consistency property does not extend to the very large variables and small number of samples. Chun and Keles (2010) formulated sparse partial least squares (SPLS) regression by relating it to sparse principal components analysis (SPCA) and developed an efficient algorithm for solving SPLS regression formulation. SPLS aims to achieve good predictive performance and variable selection by producing sparse linear combinations of the original predictors. We have used it to identify pathway regulators and found it is powerful in recognizing true regulators^80^. SPLS has two parameters: one is eta, representing the sparsity, eta should have a value between 0 and 1. The other parameters is K, which is the number of hidden (latent) components, K should take a value between 1 and min {p,(v − 1)n/v}, where p is the number of predictors and n is the sample size. We used an optimalization function to identify the optimal eta and K based on mean squared prediction errors (MSPEs) calculated for each data set and pathway. SPLS was implemented in R (https://cran.r-project.org/web/packages/spls/spls.pdf) and the R-package can be downloaded from the Comprehensive R Archive Network (CRAN)( https://cran.r-project.org/).

### Receiver operating characteristic (ROC) curves

ROC curves were plotted using R package called ggpubr (https://cran.r-project.org/web/packages/ggpubr/index.html) to investigate true positive (TP) versus false positive (FP) rate for different cut-off points in the candidate regulatory gene lists yielded from the two methods. The negative set contains all TFs except known true positive (TP) regulatory factors. For a specific list, true negative (TN) set contained all those in negative minus the false negative (FN) in the top list above the cut-off point. Each point on the ROC curve represented a true positive/false positive pair corresponding to a particular decision threshold. The sensitivity and specificity are calculated based on sensitivity = TP/(TP + FN) * 100, and specificity = TN/(TN + FP)*100.

## Supplementary Information

Supplementary Information.

## Data Availability

The R-package of TGMI can be download from http://sys.bio.mtu.edu/sample_output/TGMI/ while SPLS were adopted from CRAN library (https://cran.r-project.org/).
