# Supplementary material for: Identification of biological pathway and process regulators using sparse partial least squares and triple-gene mutual interaction
Source: Sci Rep. 2021 Jun 23;11:13174. doi: 10.1038/s41598-021-92610-4 (PMC8222328; doi:10.1038/s41598-021-92610-4)
Supplement: Supplementary file 1 — Supplementary Information. [file 41598_2021_92610_MOESM1_ESM.docx]

**Supplementary Information**

Identification of biological pathway and process regulators using sparse partial least squares and triple-gene mutual interaction

Junyan Hong^1,2^, Chathura Gunasekara^3^, Cheng He^4^, Sanzhen Liu^4^, Jianqin Huang^1,2^, Hairong Wei^5*^

^1^ School of Forestry and Biotechnology, Zhejiang Agricultural and Forestry University, Linan, Zhejiang, 311300, P.R. China

^2^ State Key Laboratory of Subtropical Silviculture, Zhejiang Agricultural and Forestry University, Linan, Zhejiang, 311300, P.R. China

^3^ Department of Pediatrics, Baylor College of Medicine, USDA/ARS Children’s Nutrition Research Center, Houston, TX 77030, USA

^4^ Department of Plant Pathology, Kansas State University, Manhattan, Kansas 66506, United States of America.

^5^ College of Forest Resources and Environmental Science, Michigan Technological University, Houghton, MI 49931, United States of America

*Correspondence to: Hairong Wei (email address: hairong@mtu.edu)

**Table S1.** Flavanone, flavonol and anthocyannin biosynthesis genes in *Arabidopsis thaliana*.

| **Gene_ID** | **Gene_name** | **Synonyms** | **Protein_function** |
| --- | --- | --- | --- |
| AT2G37040 | *PAL1* |  | Phenylalanine ammonia-lyase |
| AT3G53260 | *PAL2* |  | Phenylalanine ammonia-lyase |
| AT5G04230 | *PAL3* |  | Phenylalanine ammonia-lyase |
| AT3G10340 | *PAL4* |  | Phenylalanine ammonia-lyase |
| AT2G30490 | *C4H* |  | Cinnamic acid 4-hydroxylase |
| AT1G65060 | *4CL3* |  | 4-Coumaric acid: CoA ligase |
| AT1G36160 | *ACC1* |  | Acetyl-CoA carboxylase |
| AT5G13930 | *CHS* | *TT4* | Chalcone synthase |
| AT3G55120 | *CHI* | *TT5* | Chalcone isomerase |
| AT5G66230 | *CHI-L1* |  | Chalcone isomerase |
| AT5G05270 | *CHIL* |  | Chalcone isomerase |
| AT5G66220 |  |  | Chalcone isomerase |
| AT3G51240 | *F3H* | *TT6* | Flavanone 3-hydroxylase |
| AT5G07990 | *F3'H (CYP75B1)* | *TT7* | Flavonoid 3'-hydroxylase |
| AT5G08640 | *FLS1* |  | Flavonol synthase |
| AT5G63580 | *FLS2* |  | Flavonol synthase |
| AT5G63590 | *FLS3* |  | Flavonol synthase |
| AT5G63595 | *FLS4* |  | Flavonol synthase |
| AT5G63600 | *FLS5* |  | Flavonol synthase |
| AT5G43935 | *FLS6* |  | Flavonol synthase |
| AT5G24530 | *FNSI* | *DMR6* | Flavone synthase I activity enzyme |
| AT5G42800 | *DFR* | *TT3* | Dihydroflavonol reductase |
| AT4G22880 | *LDOX/ANS* | *TT18* | Leucoanthocyanidin dioxygenase/anthocyanidin synthase |
| AT1G61720 | *ANR* | *BAN* | Anthocyanidin reductase |
| AT5G48100 | *LAC15* | *TT10* | Laccase |
| AT1G30530 | *F3RhaT(UGT78D1)* |  | Flavonol 3-O-rhamnosyltransferase |
| AT5G17050 | *F3GlcT(UGT78D2)* |  | Flavonoid 3-O-glucosyltransferase |
| AT5G17030 | *F3AraT(UGT78D3)* |  | Flavonol 3-O-arabinosyltransferase |
| AT4G14090 | *A5GlcT(UGT75C1)* |  | Anthocyanin 5-O-glucosyltransferase |
| AT2G36790 | *F7GlcT(UGT73C6)* |  | Flavonol 7-O-glucosyltransferase |
| AT1G06000 | *F7RhaT(UGT89C1)* |  | Flavonol 7-O-rhamnosyltransferase |
| AT5G54060 | *A3Glc:2''XylT(UGT79B1)* |  | Anthocyanin 3-O-glucoside: 2"-O-xylosyltransferase |
| AT4G27830 | *BGLU10* |  | Anthocyanin 3-O-6"-O-coumaroylglucoside: glucosyltransferase |
| AT1G60270 | *BGLU6* |  | Flavonol 3-O-glucoside:6"-O-glucosyltransferase |
| AT5G54160 | *OMT1* |  | Flavonol 3'-O-methyltransferase |
| AT4G26220 | *CCOAOMT7* |  | Flavonoid O-methyltransferase |
| AT3G29590 | *5MAT* |  | Anthocyanin 5-O-glucoside:malonyltransferase |
| AT1G03940 | *A3Glc:6''CouT1* |  | Anthocyanin 3-O-glucoside: p-coumaroyltransferase |
| AT1G03495 | *A3Glc:6''CouT2* |  | Anthocyanin 3-O-glucoside: p-coumaroyltransferase |
| AT2G23000 | *SCPL10* |  | Anthocyanin sinapoyltransferase (SCPL) |
| AT2G22960 | *FPT2* |  | Flavonol phenylacyltransferase |
| AT3G59030 | *TT12* |  | Multi-drug and toxic efflux (MATE) transporter |
| AT1G17260 | *AHA10* | *TT13* | P-type H+-ATPase |
| AT4G25640 | *DTX35* |  | Multi-drug and toxic efflux (MATE) transporter |
| AT5G17220 | *GSTF12* | *TT19* | Glutathione S-transferase |

**Table S2.** Lignin biosynthesis genes in *Arabidopsis thaliana*.

| **Gene_ID** | **Gene_name** | **Protein_function** |
| --- | --- | --- |
| AT2G37040 | *PAL1* | Phenylalanine ammonia-lyase |
| AT3G53260 | *PAL2* | Phenylalanine ammonia-lyase |
| AT5G04230 | *PAL3* | Phenylalanine ammonia-lyase |
| AT3G10340 | *PAL4* | Phenylalanine ammonia-lyase |
| AT2G30490 | *C4H* | Cinnamic acid 4-hydroxylase |
| AT1G51680 | *4CL1* | 4-Coumaric acid: CoA ligase |
| AT3G21240 | *4CL2* | 4-Coumaric acid: CoA ligase |
| AT1G65060 | *4CL3* | 4-Coumaric acid: CoA ligase |
| AT3G21230 | *4CL5* | 4-Coumaric acid: CoA ligase |
| AT2G40890 | *C3H* | Coumarate 3-hydroxylase |
| AT5G48930 | *HCT* | Hydroxycinnamoyl-CoA shikimate |
| AT1G67980 | *CCOAMT* | Caffeoyl-CoA 3-O-methyltransferase |
| AT4G34050 | *CCoAOMT1* | Caffeoyl-CoA O-methyltransferase |
| AT4G26220 | *CCoAOMT7* | Caffeoyl-CoA O-methyltransferase |
| AT4G36220 | *F5H* | Ferulate 5-hydroxylase |
| AT1G15950 | *CCR1* | Cinnamoyl CoA reductase |
| AT1G80820 | *CCR2* | Cinnamoyl CoA reductase |
| AT2G33590 | *ATCRL1* | Cinnamoyl CoA reductase |
| AT1G72680 | *CAD1* | Cinnamyl alcohol dehydrogenase |
| AT2G21730 | *CAD2* | Cinnamyl alcohol dehydrogenase |
| AT2G21890 | *CAD3* | Cinnamyl alcohol dehydrogenase |
| AT3G19450 | *CAD4* | Cinnamyl alcohol dehydrogenase |
| AT4G34230 | *CAD5* | Cinnamyl alcohol dehydrogenase |
| AT4G37970 | *CAD6* | Cinnamyl alcohol dehydrogenase |
| AT4G37980 | *CAD7* | Cinnamyl alcohol dehydrogenase |
| AT4G37990 | *CAD8* | Cinnamyl alcohol dehydrogenase |
| AT4G39330 | *CAD9* | Cinnamyl alcohol dehydrogenase |
| AT1G52760 | *CSE* | Caffeoyl shikimate esterase |
| AT5G54160 | *COMT1(OMT1)* | Caffeic acid O-methyltransferase |


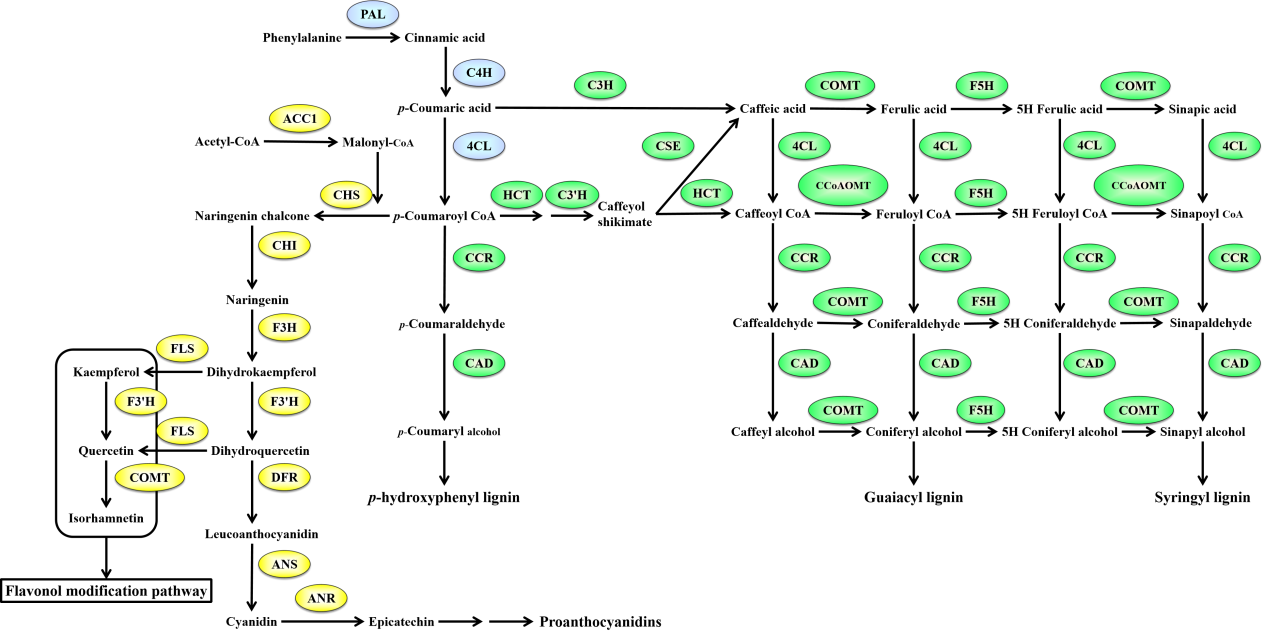


**Figure S1.** The lignin, flavanone, flavonol and anthocyannin biosynthesis pathway in *Arabidopsis thaliana.*

Phenylalanine ammonia lyase (PAL), cinnamate 4-hydroxylase (C4H), and 4-coumaryl-CoA synthetase (4CL) are the key enzymes that control the first three steps of the phenylpropanoid pathway, and thus play an important role in forging ahead the formation of downstream metabolites ^1^. Down regulation of *PAL*, *C4H* or *4CL* often significantly reduce the contents of lignin, tannin and anthocyanin in *Arabidopsis thaliana* ^2,3^. It should be noted that there are four isoforms of *4CL*, *At4CL1-At4CL3* and *At4CL5*, only *At4CL3* displays the expression and kinetic characteristics consistent with a role in flavonoid biosynthesis ^4^. The enzymes in monolignol biosynthesis also include coumarate 3-hydroxylase (C3H), hydroxycinnamoyl-CoA shikimate/Quinate hydroxycinnamoyl transferase (HCT), caffeoyl-CoA O-methyltransferase (CCoAOMT), caffeic acid /5-Hydroxyconiferyl aldehyde O-methyltransferase (OMT), ferulate 5-hydroxylase (F5H), cinnamoyl-CoA reductase (CCR), cinnamylalcohol dehydrogenase (CAD), caffeoyl shikimate esterase (CSE), and caffeic acid O-methyltransferase (COMT). *ACC1* encoding Acetyl-CoA carboxylase (ACC) is involved in the first step reaction from the phenylpropanoid pathway to the flavonoid biosynthesis ^5^. The genes encoding chalcone synthase (CHS), chalcone isomerase (CHI), flavanone 3-hydroxylase (F3H), flavonoid 3'-hydroxylase (F3'H) are the first genes discovered and defined by flavonoid biosynthesis, and they are named early biosynthetic genes (EBGs). The downstream genes are called late biosynthetic genes (LBGs), encoding proteins such as flavonol synthase (FLS), dihydroflavonol reductase (DFR), and leucoanthocyanidin dioxygenase/anthocyanidin synthase (LDOX/ANS) (Figure S1). Most mutations at LBGs can cause transparent testa (tt) mutation ^6^.

**Table S3.** Photosynthesis pathway genes in *Arabidopsis thaliana*.

| **Gene_ID** | **Gene_name** | **Protein_function** |
| --- | --- | --- |
| Light reaction pathway | | |
| Photosystem I | | |
| ATCG00350 | *psaA* | PSI-A subunit;PSI P700 apoprotein A1 |
| ATCG00340 | *psaB* | PSI-B subunit;PSI P700 apoprotein A2 |
| ATCG01060 | *psaC* | PSI-C subunit;PSI 9-kDa protein |
| AT4G02770 | *PsaD1* | PSI-D subunit |
| AT1G03130 | *PsaD2* | PSI-D subunit |
| AT4G28750 | *PsaE1* | PSI-E subunit |
| AT2G20260 | *PsaE2* | PSI-E subunit |
| AT1G31330 | *PsaF* | PSI-F subunit |
| AT1G55670 | *PsaG* | PSI-G subunit |
| AT3G16140 | *PsaH1* | PSI-H subunit |
| AT1G52230 | *PsaH2* | PSI-H subunit |
| ATCG00510 | *psaI* | PSI-I subunit |
| ATCG00630 | *psaJ* | PSI-J subunit |
| AT1G30380 | *PsaK* | PSI-K subunit |
| AT4G12800 | *PsaL* | PSI-L subunit |
| AT5G64040 | *PsaN* | PSI-N subunit |
| AT1G08380 | *PsaO* | PSI-O subunit |
| AT2G46820 | *PsaP* | PSI-P subunit |
| Photosystem II | | |
| ATCG00020 | *psbA* | PSII-A;D1 protein |
| ATCG00680 | *psbB* | PSII-B;CP47 protein |
| ATCG00280 | *psbC* | PSII-C;CP43 protein |
| ATCG00270 | *psbD* | PSII-D;D2 protein |
| ATCG00580 | *psbE* | PSII-E;Cytochrome b559 α |
| ATCG00570 | *psbF* | PSII-F;Cytochrome b559 β |
| ATCG00430 | *psbG* | PSII-G |
| ATCG00710 | *psbH* | PSII-H;10-kDa phosphoprotein |
| ATCG00080 | *psbI* | PSII-I |
| ATCG00550 | *psbJ* | PSII-J |
| ATCG00070 | *psbK* | PSII-K |
| ATCG00560 | *psbL* | PSII-L |
| ATCG00220 | *psbM* | PSII-M |
| ATCG00700 | *psbN* | PSII-N |
| AT5G66570 | *PsbO1* | PSII-O;OE33 |
| AT3G50820 | *PsbO2* | PSII-O;OE33 |
| AT1G06680 | *PsbP1* | PSII-P;OE23 |
| AT2G30790 | *PsbP2* | PSII-P;OE23 |
| AT4G21280 | *PsbQ1* | PSII-Q;OE16 |
| AT4G05180 | *PsbQ2* | PSII-Q;OE16 |
| AT1G79040 | *PsbR* | PSII-R;10-kDa polypeptide |
| AT1G44575 | *PsbS* | PSII-S;CP22 protein |
| ATCG00690 | *psbT(YCF8)* | PSII-T |
| AT2G30570 | *PsbW* | PSII-W;reaction center 6.1-kDa protein |
| AT2G06520 | *PsbX* | PSII-X |
| AT1G67740 | *PsbY(YCF32)* | PSII-Y |
| ATCG00300 | *psbZ(YCF9)* | PSII-Z |
| AT1G03600 | *Psb27* | Psb27 |
| AT4G28660 | *Psb28* | Psb28 |
| AT1G05385 | *PSB27-H1* | PSB27-H1 |
| AT5G01530 | *Lhcb4.1* | LHCII-inner CP29 |
| AT3G08940 | *Lhcb4.2* | LHCII-inner CP29 |
| AT2G40100 | *Lhcb4.3* | LHCII-inner CP29 |
| AT4G10340 | *Lhcb5* | LHCII-inner CP26 |
| AT1G15820 | *Lhcb6* | LHCII-inner CP24 |
| Intersystemic electron transport | | |
| ATCG00540 | *petA* | Cytochrome f |
| ATCG00720 | *petB* | Cytochrome b6 |
| AT4G03280 | *PetC* | Rieske protein |
| ATCG00730 | *petD* | petD |
| AT1G76100 | *PetE1* | Plastocyanin |
| AT1G20340 | *PetE2* | Plastocyanin |
| AT1G60950 | *PetF1* | Ferredoxin |
| AT1G10960 | *PetF2* | Ferredoxin |
| AT2G27510 | *PetF3* | Ferredoxin |
| AT5G10000 | *PetF4* | Ferredoxin |
| ATCG00600 | *petG* | petG |
| AT1G20020 | *PetH1* | Ferredoxin-NADP +_reductase |
| AT5G66190 | *PetH2* | Ferredoxin-NADP +_reductase |
| AT4G05390 | *PetH3* | Ferredoxin-NADP +_reductase |
| AT1G30510 | *PetH4* | Ferredoxin-NADP +_reductase |
| ATCG00590 | *petL(YCF7/ORF31)* | petL |
| AT2G26500 | *PetM* | PetM |
| ATCG00210 | *petN(YCF6)* | petN |
| ATPase | | |
| ATCG00120 | *atpA* | α-Subunit |
| ATCG00480 | *atpB* | β-Subunit |
| AT4G04640 | *AtpC1* | γ-Subunit |
| AT1G15700 | *AtpC2* | γ-Subunit |
| AT4G09650 | *AtpD* | δ-Subunit |
| ATCG00470 | *atpE* | ε-Subunit |
| ATCG00130 | *atpF* | b-Subunit |
| AT4G32260 | *AtpG* | b'-Subunit |
| ATCG00140 | *atpH* | c-Subunit |
| ATCG00150 | *atpI* | atpI |
| LHCI and LHCII | | |
| AT3G54890 | *Lhca1* | LHCI-720 |
| AT3G61470 | *Lhca2* | LHCI-680 |
| AT1G61520 | *Lhca3* | LHCI-680 |
| AT3G47470 | *Lhca4* | LHCI-720 |
| AT1G45474 | *Lhca5* | Lhca5 |
| AT1G19150 | *Lhca6* | Lhca6 |
| AT1G29920 | *Lhcb1.1* | LHCII type 1 |
| ATlG29910 | *Lhcb1.2* | LHCII type 1 |
| AT2G29930 | *Lhcb1.3* | LHCII type 1 |
| AT2G34430 | *Lhcb1.4* | LHCII type 1 |
| AT2G34420 | *Lhcb1.5* | LHCII type 1 |
| AT2G05100 | *Lhcb2.1* | LHCII type 2 |
| AT2G05070 | *Lhcb2.2* | LHCII type 2 |
| AT3G27690 | *Lhcb2.3* | LHCII type 2 |
| AT5G54270 | *Lhcb3* | LHCII type 3 |
| AT1G76570 | *Lhcb7* | LHCII type 7 |
| Calvin cycle pathway | | |
| AT1G79550 | *PGK3* | phosphoglycerate kinase |
| AT3G12780 | *PGK1* | phosphoglycerate kinase |
| AT1G56190 | *PGK2* | phosphoglycerate kinase |
| AT3G26650 | *GAPA-1* | glyceraldehyde-3-phosphate dehydrogenase |
| AT1G12900 | *GAPA-2* | glyceraldehyde-3-phosphate dehydrogenase |
| AT1G42970 | *GAPB* | glyceraldehyde-3-phosphate dehydrogenase |
| AT2G21170 | *TIM* | triose-phosphate isomerase |
| AT3G55440 | *TPI(CTIMC)* | triose-phosphate isomerase |
| AT2G21330 | *FBA1* | fructose-bisphosphate aldolase |
| AT4G38970 | *FBA2* | fructose-bisphosphate aldolase |
| AT2G01140 | *FBA3* | fructose-bisphosphate aldolase |
| AT2G36460 | *FBA6* | fructose-bisphosphate aldolase |
| AT3G52930 | *FBA8* | fructose-bisphosphate aldolase |
| AT5G03690 | *FBA4* | fructose-bisphosphate aldolase |
| AT4G26530 | *FBA5* | fructose-bisphosphate aldolase |
| AT4G26520 | *FBA7* | fructose-bisphosphate aldolase |
| AT3G55800 | *SBPASE* | sedoheptulose-1,7-bisphosphatase |
| AT3G54050 | *HCEF1* | fructose 1,6-bisphosphatase |
| AT1G43670 | *FINS1* | fructose 1,6-bisphosphatase |
| AT5G64380 |  | fructose 1,6-bisphosphatase |
| AT3G04790 | *EMB3119* | ribose-5-phosphate isomerase |
| AT5G44520 |  | ribose-5-phosphate isomerase |
| AT1G71100 | *RSW10* | ribose-5-phosphate isomerase |
| AT2G01290 | *RPI2* | ribose-5-phosphate isomerase |
| AT1G32060 | *PRK* | phosphoribulokinase |
| AT1G67090 | *RBCS1A* | ribulose-bisphosphate carboxylase |
| AT5G38410 | *RBCS3B* | ribulose bisphosphate carboxylase |
| ATCG00490 | *RBCL* | ribulose bisphosphate carboxylase |
| AT5G38430 | *RBCS1B* | ribulose bisphosphate carboxylase |
| ATMG00280 | *ORF110A* | ribulose bisphosphate carboxylase |
| AT5G38420 | *RBCS2B* | ribulose bisphosphate carboxylase |
| AT2G07732 |  | ribulose bisphosphate carboxylase |
| AT2G39730 | *RCA* | Rubisco activase |
| AT2G45290 | *TKL2* | transketolase |
| AT3G60750 | *TKL1* | transketolase |
| AT2G47400 | *CP12-1* | oxidized chloroplast protein CP12 |
| AT3G62410 | *CP12-2* | oxidized chloroplast protein CP12 |
| AT5G61410 | *RPE* | ribulose-phosphate 3-epimerase |
| AT3G01850 |  | ribulose-phosphate 3-epimerase |
| AT1G63290 |  | ribulose-phosphate 3-epimerase |


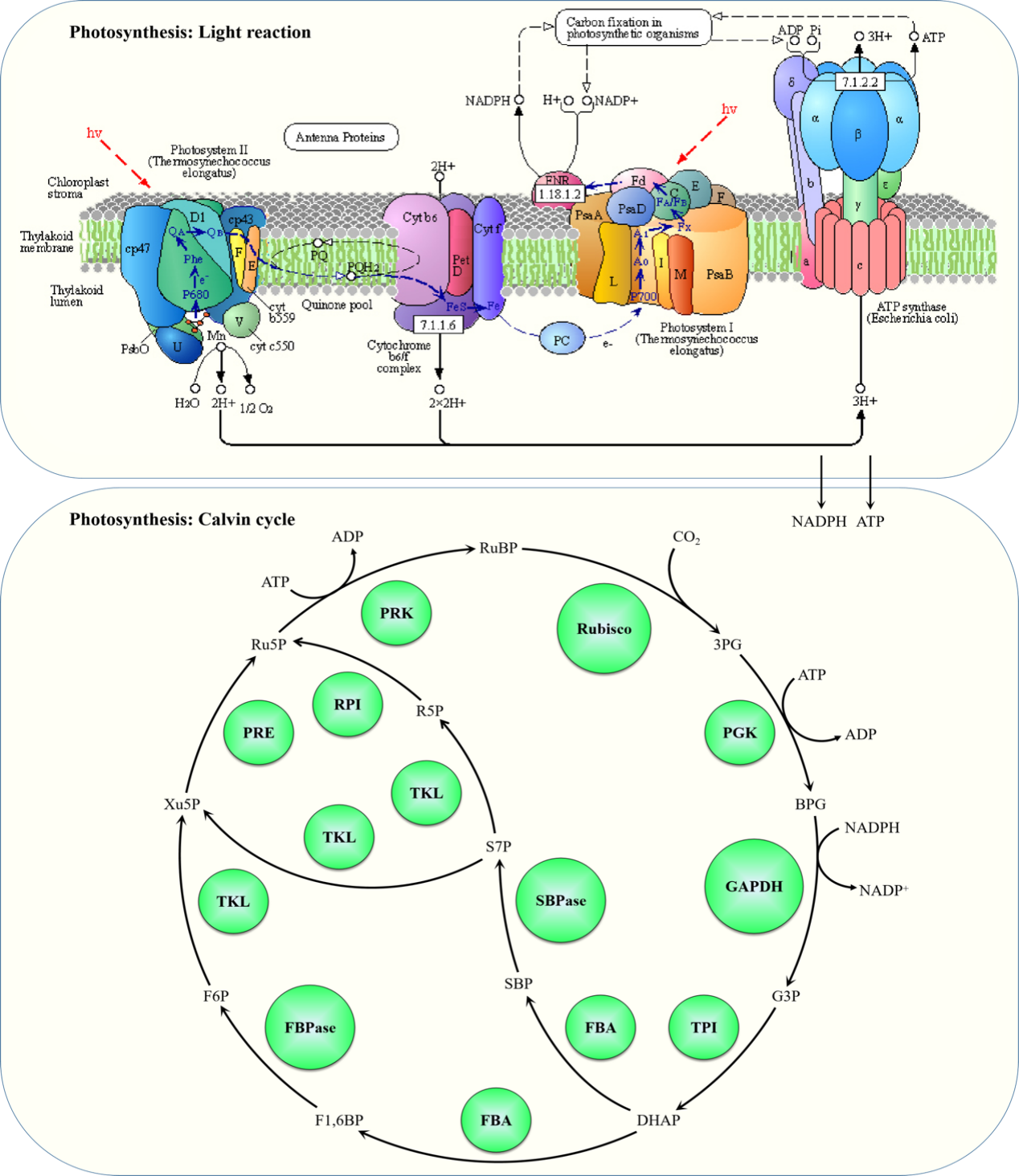


**Figure S2.** The flowchart for photosynthesis pathway in *Arabidopsis thaliana.* Upper panel: light reaction pathway (source: KEGG ^7-9^); Bottom panel: Calvin cycle pathway (the dark reaction).

Plant photosynthesis includes light reaction and dark reaction (Calvin cycle), as shown in Figure 2. The light reaction occurs on the thylakoid membrane (photosynthetic membrane) of the chloroplasts, and the light-dependent reaction provides reducing power for carbon fixation. This process involves photosystem I (PSI), photosystem II (PSII), ATP synthase complex and cytochrome b6/f complex ^10^. Photosystem I (PSI) and photosystem II (PSII) are composed of reaction centers (RCs) and tightly coupled light-harvesting complexes (LHCs) surrounding them, namely LHCI and LHCII. After absorbing light, LHCs transfer the light energy to the reaction center. After multiple steps of electron transfer, it eventually leads to oxidation of water and synthesis of NADPH and ATP ^11,12^. The PSI–LHCI complex contains a core composed of 5 chloroplast gene-encoded subunits (PsaA-C, PsaI, and PsaJ), 10 nuclear gene-encoded subunits (PsaD-H, PsaK, PsaL, PsaN, PsaO, and PsaP), and a peripheral antenna system composed of 6 LHCI proteins. *Lhca1*, *Lhca2*, *Lhca3*, and *Lhca4* encode the four main proteins of the LHCI antenna, which are assembled in dimer form, and Lhca5 and Lhca6 are the other two additional proteins in this antenna ^13,14^. PSII is the most complex multi-protein assembly in the thylakoid membrane. It contains at least 29 subunits in higher plants, such as: D1 and D2 proteins, copies of cytochrome b559, CP43 and 47. In *A. thaliana*, genes encoding these subunits include *PsbA-N*, *PsbO1*, *PsbO2*, *PsbP1*, *PsbP2*, *PsbQ1*, *PsbQ2*, *PsbR-T*, *PsbW-Z*, *Psb27*, *Psb27**-H1* and *Psb28*; among these genes, *PsbA-N*, *PsbT* and *PsbZ* are chloroplast genome genes. *Lhcb1.1-1.5*, *Lhcb2.1-2.3* and *Lhcb3*, the three major proteins of LHCII antenna, form heterotimers and constitute the outermost layer of PSII antenna proteins. Monomer CP29, cp26 and Cp24, the secondary proteins of LHCII antenna, are encoded by *Lhcb4.1-4.3*, *Lhcb5* and *Lhcb6,* respectively ^15^. In addition, the *Lhcb7* gene of *A. thaliana* has also been reported ^16^.

The NADPH and ATP generated by the light reaction are subsequently used by Calvin cycle to convert CO_2_ into sugars and other organic substances. Of the 11 enzymes in the Calvin cycle, five are phosphoglycerate kinase (PGK), glyceraldehyde 3-phosphatedehydrogenase (GAPDH) , triose phosphate isomerase (TPI), fructose bisphosphate aldolase (FBA), and fructose-1,6-bisphosphatase (FBPase), three genes encoding transketolase (TKL), ribulose-5-phosphate 3-epimerase (RPE), and ribose-5-phosphateisomerase (RPI) are from the non-oxidative pentose phosphate pathway and three genes encoding asedoheptulose-1,7-bisphosphatase (SBPase), phosphoribulokinase (PRK), and ribulose-1,5-bisphosphate carboxylase/oxygenase (Rubisco), are unique to photosynthesis ^17,18^.

**Table S4.** Lignin pathway genes in *Populus trichocarpa*.

| **locusName** | **Best_arabi_gene** | **AT_symbol** | **Ptr_symbol** |  | **Protein function** |
| --- | --- | --- | --- | --- | --- |
| Potri.006G126800 | AT3G53260 | *PAL2* | *PAL1* | *PtrPAL1* | Phenylalanine ammonia-lyase |
| Potri.008G038200 |  |  | *PAL2* | *PtrPAL2* | Phenylalanine ammonia-lyase |
| Potri.016G091100 | AT3G53260 | *PAL2* | *PAL3* | *PtrPAL3* | Phenylalanine ammonia-lyase |
| Potri.010G224100 |  |  | *PAL4* | *PtrPAL4* | Phenylalanine ammonia-lyase |
| Potri.010G224200 |  |  | *PAL5* | *PtrPAL5* | Phenylalanine ammonia-lyase |
| Potri.013G157900 | AT2G30490 | *C4H* | *C4H1* | *PtrC4H1* | Cinnamic acid 4-hydroxylase |
| Potri.019G130700 | AT2G30490 | *C4H* | *C4H2* | *PtrC4H2* | Cinnamic acid 4-hydroxylase |
| Potri.001G036900 | AT1G51680 | *4CL1* | *4CL3* | *Ptr4CL3* | 4-Coumaric acid: CoA ligase |
| Potri.019G049500 | AT1G65060 | *4CL3* | *4CL4* | *Ptr4CL4* | 4-Coumaric acid: CoA ligase |
| Potri.003G188500 | AT1G51680 | *4CL1* | *4CL5* | *Ptr4CL5* | 4-Coumaric acid: CoA ligase |
| Potri.018G094200 | AT1G51680 | *4CL1* |  | *At4CL1_Potri.018G094200* | 4-Coumaric acid: CoA ligase |
| Potri.006G169700 | AT1G51680 | *4CL1* |  | *At4CL1_Potri.006G169700* | 4-Coumaric acid: CoA ligase |
| Potri.006G033300 | AT2G40890 | *C3H* | *C3H3* | *PtrC3H3* | Coumarate 3-hydroxylase |
| Potri.003G183900 | AT5G48930 | *HCT* | *HCT1* | *PtrHCT1* | Hydroxycinnamoyl-CoA shikimate |
| Potri.001G042900 | AT5G48930 | *HCT* | *HCT6* | *PtrHCT6* | Hydroxycinnamoyl-CoA shikimate |
| Potri.009G099800 | AT4G34050 | *CCoAOMT1* | *CCoAOMT1* | *PtrCCoAOMT1* | Caffeoyl-CoA O-methyltransferase |
| Potri.001G304800 | AT4G34050 | *CCoAOMT1* | *CCoAOMT2* | *PtrCCoAOMT2* | Caffeoyl-CoA O-methyltransferase |
| Potri.008G136600 |  |  | *CCoAOMT3* | *PtrCCoAOMT3* | Caffeoyl-CoA O-methyltransferase |
| Potri.018G070300 | AT4G26220 | *CCoAOMT7* |  | *AtCCoAOMT7_Potri.018G070300* | Caffeoyl-CoA O-methyltransferase |
| Potri.005G117500 | AT4G36220 | *F5H* | *CAld5H1,F5H1* | *PtrF5H1* | Ferulate 5-hydroxylase |
| Potri.007G016400 | AT4G36220 | *F5H* | *CAld5H2,F5H2* | *PtrF5H2* | Ferulate 5-hydroxylase |
| Potri.003G181400 | AT1G80820 | *CCR2* | *CCR2* | *PtrCCR2* | Cinnamoyl CoA reductase |
| Potri.001G045000 | AT1G80820 | *CCR2* |  | *AtCCR2_Potri.001G045000* | Cinnamoyl CoA reductase |
| Potri.001G045100 | AT1G80820 | *CCR2* |  | *AtCCR2_Potri.001G045100* | Cinnamoyl CoA reductase |
| Potri.001G045500 | AT1G80820 | *CCR2* |  | *AtCCR2_Potri.001G045500* | Cinnamoyl CoA reductase |
| Potri.001G046100 | AT1G80820 | *CCR2* |  | *AtCCR2_Potri.001G046100* | Cinnamoyl CoA reductase |
| Potri.001G046400 | AT1G80820 | *CCR2* |  | *AtCCR2_Potri.001G046400* | Cinnamoyl CoA reductase |
| Potri.009G095800 | AT3G19450 | *CAD4* | *CAD1* | *PtrCAD1* | Cinnamyl alcohol dehydrogenase |
| Potri.016G078300 | AT4G37970 | *CAD6* | *CAD2* | *PtrCAD2* | Cinnamyl alcohol dehydrogenase |
| Potri.001G307200 | AT4G39330 | *CAD9* |  | *AtCAD9_Potri.001G307200* | Cinnamyl alcohol dehydrogenase |
| Potri.001G268600 | AT4G37990 | *ATCAD8* |  | *AtCAD8_Potri.001G268600* | Cinnamyl alcohol dehydrogenase |
| Potri.006G199100 | AT4G37990 | *ATCAD8* |  | *AtCAD8_Potri.006G199100* | Cinnamyl alcohol dehydrogenase |
| Potri.009G063400 | AT4G37990 | *ATCAD8* |  | *AtCAD8_Potri.009G063400* | Cinnamyl alcohol dehydrogenase |
| Potri.016G065300 | AT4G37990 | *ATCAD8* |  | *AtCAD8_Potri.016G065300* | Cinnamyl alcohol dehydrogenase |
| Potri.011G148100 | AT1G72680 | *CAD1* |  | *AtCAD1_Potri.011G148100* | Cinnamyl alcohol dehydrogenase |
| Potri.011G148200 | AT1G72680 | *CAD1* |  | *AtCAD1_Potri.011G148200* | Cinnamyl alcohol dehydrogenase |
| Potri.003G059200 | AT1G52760 | *CSE* | *CSE1* | *PtrCSE1* | Caffeoyl shikimate esterase |
| Potri.001G175000 | AT1G52760 | *CSE* | *CSE2* | *PtrCSE2* | Caffeoyl shikimate esterase |
| Potri.015G003100 | AT5G54160 | *COMT1* | *COMT1* | *PtrCOMT1* | Caffeic acid O-methyltransferase |
| Potri.012G006400 | AT5G54160 | *COMT1* | *COMT2* | *PtrCOMT2* | Caffeic acid O-methyltransferase |

**Table S5.** Regulatory genes identified and ranked by TGMI and SPLS for unified pathway of flavanone, flavonol and anthocyannin biosyntheisis in *Arabidopsis thaliana*. Only the top 50 transcription factors that have the highest connectivity to pathway genes are shown.

| **TGMI** | | | |  | **SPLS** | | | |
| --- | --- | --- | --- | --- | --- | --- | --- | --- |
| **Rank** | **TF** | **Symbol** | **Freq.** |  | **Rank** | **TF** | **Symbol** | **Freq.** |
| **1** | **AT1G54160** | **NFYA5^19^** | **23** |  | **1** | **AT2G22540** | **SVP^20^** | **32** |
| **2** | **AT1G04250** | **IAA17** | **21** |  | **2** | **AT1G72830** | **NF-YA3** | **30** |
| **3** | **AT1G67310** | **---** | **17** |  | **3** | **AT1G77850** | **ARF17** | **30** |
| **4** | **AT5G48670** | **FEM111** | **17** |  | **4** | **AT3G18400** | **NAC058** | **30** |
| **5** | **AT1G05805** | **---** | **16** |  | **5** | **AT3G61910** | **NST2** | **30** |
| **6** | **AT3G20910** | **NF-YA9^21^** | **16** |  | **6** | **AT4G21750** | **ATML1** | **30** |
| **7** | **AT4G00480** | **MYC1^22^** | **16** |  | **7** | **AT4G28500** | **SND2** | **30** |
| **8** | **AT5G60450** | **ARF4** | **16** |  | **8** | **AT2G33810** | **SPL3** | **29** |
| **9** | **AT2G14210** | **ANR1** | **15** |  | **9** | **AT2G38250** | **---** | **29** |
| **10** | **AT2G41710** | **---** | **15** |  | **10** | **AT2G44745** | **WRKY12** | **28** |
| **11** | **AT5G01200** | **---** | **15** |  | **11** | **AT1G17590** | **NF-YA8** | **27** |
| **12** | **AT5G65410** | **ZHD1** | **15** |  | **12** | **AT1G28470** | **SND3** | **27** |
| **13** | **AT1G01720** | **ATAF1^23^** | **14** |  | **13** | **AT1G32770** | **SND1** | **27** |
| **14** | **AT1G16070** | **TLP8** | **14** |  | **14** | **AT1G34310** | **ARF12** | **27** |
| **15** | **AT2G22540** | **SVP^20^** | **14** |  | **15** | **AT1G79430** | **WDY** | **27** |
| **16** | **AT3G14020** | **NF-YA6** | **14** |  | **16** | **AT2G19810** | **OZF1** | **27** |
| **17** | **AT3G14740** | **---** | **14** |  | **17** | **AT2G40470** | **LBD15** | **27** |
| **18** | **AT3G15510** | **NARS1^24^** | **14** |  | **18** | **AT5G20900** | **TIFY3B** | **27** |
| **19** | **AT3G42790** | **AL3** | **14** |  | **19** | **AT1G05805** | **---** | **26** |
| **20** | **AT3G60030** | **SPL12** | **14** |  | **20** | **AT1G48000** | **MYB112^25^** | **26** |
| **21** | **AT4G11080** | **3xHMG-box1** | **14** |  | **21** | **AT4G14770** | **TCX2** | **26** |
| **22** | **AT1G28520** | **VOZ1** | **13** |  | **22** | **AT4G24660** | **ZHD2** | **26** |
| **23** | **AT1G75540** | **STH2^26^** | **13** |  | **23** | **AT5G47790** | **---** | **26** |
| **24** | **AT1G76590** | **---** | **13** |  | **24** | **AT5G60410** | **SIZ1** | **26** |
| **25** | **AT2G21530** | **---** | **13** |  | **25** | **AT2G17040** | **NAC036** | **25** |
| **26** | **AT2G33310** | **IAA13** | **13** |  | **26** | **AT2G47460** | **PFG1/MYB12^27^** | **25** |
| **27** | **AT3G12250** | **TGA6** | **13** |  | **27** | **AT3G01140** | **NOK** | **25** |
| **28** | **AT4G12620** | **UNE13** | **13** |  | **28** | **AT3G23240** | **ERF1** | **25** |
| **29** | **AT4G23800** | **3xHMG-box2** | **13** |  | **29** | **AT4G11080** | **3xHMG-box1** | **25** |
| **30** | **AT4G24660** | **ZHD2** | **13** |  | **30** | **AT5G02030** | **VAN** | **25** |
| **31** | **AT4G29100** | **---** | **13** |  | **31** | **AT5G15150** | **HB-3** | **25** |
| **32** | **AT4G34990** | **MYB32^28^** | **13** |  | **32** | **AT5G24800** | **BZO2H2** | **25** |
| **33** | **AT5G64530** | **XND1** | **13** |  | **33** | **AT5G65410** | **ZHD1** | **25** |
| **34** | **AT2G40950** | **BZIP17** | **12** |  | **34** | **AT1G14580** | **---** | **24** |
| **35** | **AT3G06740** | **GATA15** | **12** |  | **35** | **AT1G16490** | **MYB58** | **24** |
| **36** | **AT3G17609** | **HYH^29^** | **12** |  | **36** | **AT1G27370** | **SPL10** | **24** |
| **37** | **AT4G21550** | **VAL3** | **12** |  | **37** | **AT1G31320** | **LBD4** | **24** |
| **38** | **AT5G12840** | **NF-YA1^21^** | **12** |  | **38** | **AT1G56650** | **PAP1/MYB75^27^** | **24** |
| **39** | **AT1G43770** | **---** | **11** |  | **39** | **AT1G63910** | **MYB103** | **24** |
| **40** | **AT1G52890** | **NAC019^30^** | **11** |  | **40** | **AT1G66810** | **C3H14** | **24** |
| **41** | **AT1G71130** | **CRF8** | **11** |  | **41** | **AT2G35310** | **---** | **24** |
| **42** | **AT1G72830** | **NF-YA3** | **11** |  | **42** | **AT3G07340** | **---** | **24** |
| **43** | **AT1G76350** | **---** | **11** |  | **43** | **AT3G49930** | **---** | **24** |
| **44** | **AT1G77920** | **---** | **11** |  | **44** | **AT4G01680** | **MYB55** | **24** |
| **45** | **AT2G04890** | **SCL21** | **11** |  | **45** | **AT4G35900** | **FD-1** | **24** |
| **46** | **AT2G18350** | **ZHD6** | **11** |  | **46** | **AT5G25830** | **GATA12** | **24** |
| **47** | **AT2G35940** | **EDA29** | **11** |  | **47** | **AT5G38140** | **NF-YC12** | **24** |
| **48** | **AT3G11440** | **MYB65^31^** | **11** |  | **48** | **AT5G57390** | **PLT5** | **24** |
| **49** | **AT3G54220** | **SGR1** | **11** |  | **49** | **AT5G62165** | **FYF** | **24** |
| **50** | **AT3G62090** | **PIL2** | **11** |  | **50** | **AT5G66940** | **---** | **24** |

*The TF marked with red color on the yellow background is a known regulatory factor of this pathway. Frequency represents the number of genes regulated by each TF in the pathway.

**Table S6.** Regulatory genes identified and ranked by TGMI and SPLS for lignin pathway in *Arabidopsis thaliana*. Only the top 50 transcription factors that have the highest connectivity to pathway genes are shown.

| **TGMI** | | | |  | **SPLS** | | | |
| --- | --- | --- | --- | --- | --- | --- | --- | --- |
| **Rank** | **ID** | **Symbol** | **Freq.** |  | **Rank** | **ID** | **Symbol** | **Freq.** |
| **1** | **AT4G22680** | **MYB85^32^** | **13** |  | **1** | **AT2G41690** | **HSFB3** | **26** |
| **2** | **AT1G32770** | **SND1^33^** | **12** |  | **2** | **AT2G46770** | **NST1^33^** | **26** |
| **3** | **AT3G61910** | **NST2^33^** | **11** |  | **3** | **AT4G21750** | **ATML1** | **26** |
| **4** | **AT1G16490** | **MYB58^34^** | **9** |  | **4** | **AT4G36990** | **HSFB1** | **26** |
| **5** | **AT1G28470** | **SND3^35^** | **9** |  | **5** | **AT1G13600** | **bZIP58** | **25** |
| **6** | **AT1G79180** | **MYB63^34^** | **9** |  | **6** | **AT2G22850** | **bZIP6^36^** | **25** |
| **7** | **AT2G46770** | **NST1^33^** | **9** |  | **7** | **AT2G25000** | **WRKY60** | **25** |
| **8** | **AT2G40470** | **LBD15^37^** | **8** |  | **8** | **AT4G00220** | **LBD30^38^** | **25** |
| **9** | **AT5G16600** | **MYB43^32^** | **8** |  | **9** | **AT4G31420** | **---** | **25** |
| **10** | **AT1G63910** | **MYB103^39^** | **7** |  | **10** | **AT5G12850** | **---** | **25** |
| **11** | **AT1G52150** | **ICU4** | **6** |  | **11** | **AT5G60410** | **SIZ1^38^** | **25** |
| **12** | **AT5G12870** | **MYB46^40^** | **6** |  | **12** | **AT1G17950** | **MYB52^41^** | **24** |
| **13** | **AT5G60450** | **ARF4** | **6** |  | **13** | **AT1G74650** | **MYB31^42^** | **24** |
| **14** | **AT1G09540** | **MYB61^41^** | **5** |  | **14** | **AT2G47070** | **SPL1** | **24** |
| **15** | **AT1G68200** | **C3H15^43^** | **5** |  | **15** | **AT4G22680** | **MYB85^32^** | **24** |
| **16** | **AT2G33310** | **IAA13** | **5** |  | **16** | **AT4G28500** | **SND2^35^** | **24** |
| **17** | **AT2G44745** | **WRKY12^44^** | **5** |  | **17** | **AT5G15840** | **FG** | **24** |
| **18** | **AT5G25890** | **IAR2** | **5** |  | **18** | **AT1G04100** | **IAA10** | **23** |
| **19** | **AT1G19520** | **NFD5** | **4** |  | **19** | **AT1G12260** | **VND4^45^** | **23** |
| **20** | **AT4G32010** | **VAL2** | **4** |  | **20** | **AT1G14410** | **WHY1** | **23** |
| **21** | **AT4G37790** | **HAT22** | **4** |  | **21** | **AT1G32770** | **SND1^33^** | **23** |
| **22** | **AT1G31470** | **NFD4** | **3** |  | **22** | **AT1G33240** | **GTL1** | **23** |
| **23** | **AT1G69170** | **---** | **3** |  | **23** | **AT1G63910** | **MYB103^39^** | **23** |
| **24** | **AT2G14210** | **ANR1** | **3** |  | **24** | **AT1G66810** | **C3H14^43^** | **23** |
| **25** | **AT2G37650** | **---** | **3** |  | **25** | **AT1G74660** | **MIF1** | **23** |
| **26** | **AT2G41690** | **HSFB3** | **3** |  | **26** | **AT1G76110** | **---** | **23** |
| **27** | **AT2G41710** | **---** | **3** |  | **27** | **AT1G79180** | **MYB63^34^** | **23** |
| **28** | **AT2G41900** | **OXS2** | **3** |  | **28** | **AT2G38250** | **---** | **23** |
| **29** | **AT3G54430** | **SRS6** | **3** |  | **29** | **AT2G46310** | **CRF5** | **23** |
| **30** | **AT4G28500** | **SND2^35^** | **3** |  | **30** | **AT3G53370** | **---** | **23** |
| **31** | **AT4G33450** | **MYB69^41^** | **3** |  | **31** | **AT3G61910** | **NST2^33^** | **23** |
| **32** | **AT5G26660** | **MYB86^28^** | **3** |  | **32** | **AT4G34610** | **BLH6^46^** | **23** |
| **33** | **AT5G48560** | **---** | **3** |  | **33** | **AT5G04940** | **SUVH1** | **23** |
| **34** | **AT5G60410** | **SIZ1^38^** | **3** |  | **34** | **AT5G06960** | **TGA5** | **23** |
| **35** | **AT5G62000** | **ARF2** | **3** |  | **35** | **AT5G16470** | **---** | **23** |
| **36** | **AT5G64530** | **XND1^47^** | **3** |  | **36** | **AT5G48560** | **---** | **23** |
| **37** | **AT1G04550** | **IAA12** | **2** |  | **37** | **AT5G57520** | **ZFP2** | **23** |
| **38** | **AT1G14410** | **WHY1** | **2** |  | **38** | **AT1G12860** | **SCRM2** | **22** |
| **39** | **AT1G30330** | **ARF6** | **2** |  | **39** | **AT1G22590** | **AGL87** | **22** |
| **40** | **AT1G67310** | **---** | **2** |  | **40** | **AT1G50640** | **ERF3** | **22** |
| **41** | **AT1G80730** | **ZFP1** | **2** |  | **41** | **AT1G62300** | **WRKY6** | **22** |
| **42** | **AT2G22850** | **bZIP6^36^** | **2** |  | **42** | **AT1G62990** | **KNAT7^46^** | **22** |
| **43** | **AT2G37120** | **---** | **2** |  | **43** | **AT1G68200** | **C3H15^43^** | **22** |
| **44** | **AT2G40950** | **BZIP17^36^** | **2** |  | **44** | **AT1G74950** | **TIFY10B** | **22** |
| **45** | **AT3G23050** | **IAA7** | **2** |  | **45** | **AT1G80730** | **ZFP1** | **22** |
| **46** | **AT3G55770** | **WLIM2b** | **2** |  | **46** | **AT2G02070** | **IDD5** | **22** |
| **47** | **AT3G61890** | **HB-12** | **2** |  | **47** | **AT2G20180** | **PIL5** | **22** |
| **48** | **AT5G25830** | **GATA12^48^** | **2** |  | **48** | **AT4G33450** | **MYB69^41^** | **22** |
| **49** | **AT5G46760** | **MYC3** | **2** |  | **49** | **AT5G12870** | **MYB46^40^** | **22** |
| **50** | **AT5G60690** | **REV^46^** | **2** |  | **50** | **AT5G16600** | **MYB43^32^** | **22** |

*The TF marked with red color on the yellow background is a known regulatory factor of this pathway. Frequency represents the number of genes regulated by each TF in the pathway. The superscript number of each gene symbol represents the reference numbers.

**Table S7.** Regulatory genes identified and ranked by TGMI and SPLS for lignin pathway in *Populus trichocarpa*.

Only the top 50 transcription factors that have the highest connectivity to pathway genes are shown.

| **TGMI** | | | | |  | **SPLS** | | | | |
| --- | --- | --- | --- | --- | --- | --- | --- | --- | --- | --- |
| **Rank** | **ID** | **Best_At_ID** | **Symbol** | **Freq.** |  | **Rank** | **ID** | **Best_At_ID** | **Symbol** | **Freq.** |
| **1** | **Potri.007G014400** | **AT4G36160** | **VND2^45^** | **19** |  | **1** | **Potri.017G016700** | **AT4G28500** | **SND2^35^** | **24** |
| **2** | **Potri.007G135300** | **AT4G28500** | **SND2^35^** | **19** |  | **2** | **Potri.007G014400** | **AT4G36160** | **VND2^45^** | **23** |
| **3** | **Potri.016G056500** | **AT2G41690** | **HSFB3** | **19** |  | **3** | **Potri.007G135300** | **AT4G28500** | **SND2^35^** | **21** |
| **4** | **Potri.001G118800** | **AT4G22680** | **MYB85^32^** | **18** |  | **4** | **Potri.016G056500** | **AT2G41690** | **HSFB3** | **20** |
| **5** | **Potri.002G138900** | **AT2G44745** | **WRKY12^44^** | **18** |  | **5** | **Potri.006G049200** | **AT2G41690** | **HSFB3** | **19** |
| **6** | **Potri.003G114100** | **AT4G22680** | **MYB85^32^** | **18** |  | **6** | **Potri.008G025600** | **AT3G10000** | **EDA31** | **19** |
| **7** | **Potri.004G203400** | **AT1G08320** | **TGA9** | **18** |  | **7** | **Potri.017G130300** | **AT5G16600** | **MYB43^32^** | **19** |
| **8** | **Potri.006G049200** | **AT2G41690** | **HSFB3** | **18** |  | **8** | **Potri.001G118800** | **AT4G22680** | **MYB85^32^** | **18** |
| **9** | **Potri.007G138300** | **AT5G44260** | **---** | **18** |  | **9** | **Potri.001G420400** | **AT3G20550** | **DDL** | **17** |
| **10** | **Potri.017G016700** | **AT4G28500** | **SND2^35^** | **18** |  | **10** | **Potri.005G063200** | **AT4G33450** | **MYB69^41^** | **17** |
| **11** | **Potri.017G130300** | **AT5G16600** | **MYB43^32^** | **18** |  | **11** | **Potri.008G041900** | **AT3G52250** | **---** | **16** |
| **12** | **Potri.002G149000** | **AT2G45420** | **LBD18^49^** | **16** |  | **12** | **Potri.002G113300** | **AT4G08150** | **KNAT1^50^** | **15** |
| **13** | **Potri.003G090300** | **AT1G32540** | **LOL1** | **16** |  | **13** | **Potri.010G223300** | **AT3G54810** | **GATA8** | **15** |
| **14** | **Potri.005G063200** | **AT4G33450** | **MYB69^41^** | **16** |  | **14** | **Potri.011G059300** | **AT2G33860** | **ETT** | **14** |
| **15** | **Potri.007G106100** | **AT4G33450** | **MYB69^41^** | **16** |  | **15** | **Potri.012G100700** | **AT5G50670** | **SPL13B** | **14** |
| **16** | **Potri.012G100700** | **AT5G50670** | **SPL13B** | **16** |  | **16** | **Potri.010G167901** | **AT1G69580** | **---** | **11** |
| **17** | **Potri.005G129500** | **AT2G23760** | **BLH4^51^** | **15** |  | **17** | **Potri.003G090300** | **AT1G32540** | **LOL1** | **10** |
| **18** | **Potri.010G223300** | **AT3G54810** | **GATA8** | **15** |  | **18** | **Potri.014G051200** | **AT2G44730** | **---** | **9** |
| **19** | **Potri.014G070400** | **AT2G45420** | **LBD18^49^** | **15** |  | **19** | **Potri.002G176300** | **AT3G61890** | **HB-12** | **8** |
| **20** | **Potri.015G014000** | **AT4G27900** | **---** | **15** |  | **20** | **Potri.005G117300** | **AT3G51120** | **---** | **8** |
| **21** | **Potri.016G136500** | **AT4G32010** | **VAL2** | **15** |  | **21** | **Potri.012G006100** | **AT5G24520** | **URM23** | **8** |
| **22** | **Potri.017G082900** | **AT3G28920** | **ZHD9** | **15** |  | **22** | **Potri.013G086400** | **AT5G16470** | **---** | **8** |
| **23** | **Potri.002G257800** | **AT4G28610** | **PHR1** | **14** |  | **23** | **Potri.016G065400** | **AT5G06550** | **---** | **8** |
| **24** | **Potri.006G152700** | **AT4G29230** | **NAC075^52^** | **14** |  | **24** | **Potri.001G392801** | **AT3G14980** | **---** | **6** |
| **25** | **Potri.007G039500** | **AT5G66870** | **LBD36** | **14** |  | **25** | **Potri.011G091900** | **AT1G30330** | **ARF6** | **6** |
| **26** | **Potri.010G039233** | **AT1G70000** | **---** | **14** |  | **26** | **Potri.014G025100** | **AT5G52010** | **---** | **6** |
| **27** | **Potri.018G068700** | **AT4G29230** | **NAC075^52^** | **14** |  | **27** | **Potri.014G111700** | **AT2G46990** | **IAA20** | **6** |
| **28** | **Potri.001G099800** | **AT1G63910** | **MYB103^39^** | **13** |  | **28** | **Potri.015G089100** | **AT3G48440** | **---** | **6** |
| **29** | **Potri.005G134900** | **AT5G66870** | **LBD36** | **13** |  | **29** | **Potri.016G128300** | **AT2G38470** | **WRKY33** | **6** |
| **30** | **Potri.006G259400** | **AT5G11060** | **KNAT4** | **13** |  | **30** | **Potri.006G279100** | **AT4G31270** | **---** | **5** |
| **31** | **Potri.009G064500** | **AT5G26880** | **AGL26** | **13** |  | **31** | **Potri.015G061900** | **AT5G61380** | **TOC1** | **5** |
| **32** | **Potri.016G018600** | **AT2G36450** | **HRD** | **13** |  | **32** | **Potri.017G072460** | **AT3G01460** | **MBD9** | **5** |
| **33** | **Potri.003G065400** | **AT3G13840** | **---** | **12** |  | **33** | **Potri.003G168900** | **AT5G41020** | **---** | **4** |
| **34** | **Potri.007G032700** | **AT2G23760** | **BLH4^51^** | **12** |  | **34** | **Potri.004G048675** | **AT1G28420** | **HB-1** | **4** |
| **35** | **Potri.008G097900** | **AT1G69170** | **---** | **12** |  | **35** | **Potri.010G098200** | **AT2G02160** | **---** | **4** |
| **36** | **Potri.009G164300** | **AT1G08320** | **TGA9** | **12** |  | **36** | **Potri.014G156300** | **AT4G15420** | **---** | **4** |
| **37** | **Potri.010G193000** | **AT3G11280** | **---** | **12** |  | **37** | **Potri.018G064500** | **AT3G43240** | **---** | **4** |
| **38** | **Potri.012G126500** | **AT1G62700** | **VND5^45^** | **12** |  | **38** | **Potri.003G028245** | **AT1G15790** | **---** | **3** |
| **39** | **Potri.013G081200** | **AT2G40470** | **LBD15^37^** | **12** |  | **39** | **Potri.007G135500** | **AT5G44180** | **---** | **3** |
| **40** | **Potri.002G096800** | **AT1G34670** | **MYB93** | **11** |  | **40** | **Potri.008G045600** | **AT2G20280** | **---** | **3** |
| **41** | **Potri.010G209400** | **AT2G28710** | **---** | **11** |  | **41** | **Potri.008G161100** | **AT1G04240** | **SHY2** | **3** |
| **42** | **Potri.015G033600** | **AT1G73410** | **MYB54^41^** | **11** |  | **42** | **Potri.009G125400** | **AT4G38900** | **---** | **3** |
| **43** | **Potri.003G113000** | **AT1G62700** | **VND5^45^** | **10** |  | **43** | **Potri.018G107000** | **AT4G30935** | **WRKY32** | **3** |
| **44** | **Potri.003G132000** | **AT1G63910** | **MYB103^39^** | **10** |  | **44** | **Potri.001G163700** | **AT3G14230** | **RAP2.2** | **2** |
| **45** | **Potri.009G149400** | **AT3G44750** | **HDT1** | **10** |  | **45** | **Potri.002G125400** | **AT1G45249** | **ATAREB1** | **2** |
| **46** | **Potri.011G059300** | **AT2G33860** | **ETT** | **10** |  | **46** | **Potri.002G237950** | **AT5G13960** | **SUVH4** | **2** |
| **47** | **Potri.002G157300** | **AT1G01780** | **PLIM2b** | **9** |  | **47** | **Potri.004G124000** | **AT3G28730** | **SSRP1** | **2** |
| **48** | **Potri.002G239400** | **AT4G12850** | **---** | **9** |  | **48** | **Potri.005G114700** | **AT2G17950** | **WUS1** | **2** |
| **49** | **Potri.014G066100** | **AT3G60580** | **---** | **9** |  | **49** | **Potri.006G075500** | **AT4G29940** | **PRHA** | **2** |
| **50** | **Potri.002G113300** | **AT4G08150** | **KNAT1^50^** | **8** |  | **50** | **Potri.007G109100** | **AT5G09330** | **VNI1** | **2** |

*The TF marked with red color on the yellow background is a known regulatory factor of this pathway. Frequency represents the number of genes regulated by each TF in the pathway.

**Table S8.** Regulatory genes identified and ranked by TGMI and SPLS for unified pathway of Light reaction and Calvin cycle in *Arabidopsis thaliana.* Only the top 50 transcription factors that have the highest connectivity to pathway genes are shown.

| **TGMI** | | | |  | **SPLS** | | | |
| --- | --- | --- | --- | --- | --- | --- | --- | --- |
| **Rank** | **ID** | **Symbol** | **Freq.** |  | **Rank** | **ID** | **Symbol** | **Freq.** |
| **1** | **AT1G06040** | **STO/BBX24^53^** | **43** |  | **1** | **AT3G50750** | **BEH1^54^** | **112** |
| **2** | **AT2G45190** | **YAB1** | **40** |  | **2** | **AT3G59060** | **PIF5^55^** | **111** |
| **3** | **AT3G16350** | **---** | **38** |  | **3** | **AT3G58630** | **---** | **110** |
| **4** | **AT1G73870** | **COL7^56^** | **37** |  | **4** | **AT5G57660** | **COL5^57^** | **109** |
| **5** | **AT3G47500** | **CDF3** | **34** |  | **5** | **AT3G20770** | **EIN3^58^** | **108** |
| **6** | **AT3G59060** | **PIF5^55^** | **32** |  | **6** | **AT4G35580** | **NTL9** | **108** |
| **7** | **AT1G34180** | **NAC016^59^** | **31** |  | **7** | **AT5G16680** | **---** | **108** |
| **8** | **AT3G60110** | **---** | **31** |  | **8** | **AT3G23050** | **IAA7** | **107** |
| **9** | **AT5G15160** | **BNQ2^57^** | **31** |  | **9** | **AT4G28610** | **PHR1** | **107** |
| **10** | **AT5G61270** | **PIF7^60^** | **30** |  | **10** | **AT5G63420** | **EMB2746** | **107** |
| **11** | **AT4G30860** | **SDG4** | **29** |  | **11** | **AT5G66730** | **IDD1** | **107** |
| **12** | **AT2G31380** | **STH/BBX25^61^** | **28** |  | **12** | **AT1G08620** | **PKDM7D** | **106** |
| **13** | **AT5G65210** | **TGA1** | **28** |  | **13** | **AT2G46810** | **MYC70** | **105** |
| **14** | **AT1G04250** | **IAA17** | **27** |  | **14** | **AT5G35210** | **PTM** | **105** |
| **15** | **AT1G47870** | **E2FC** | **27** |  | **15** | **AT1G28520** | **VOZ1** | **104** |
| **16** | **AT2G43010** | **PIF4^55^** | **27** |  | **16** | **AT2G25180** | **RR12** | **104** |
| **17** | **AT5G48250** | **BBX8** | **27** |  | **17** | **AT3G52250** | **---** | **104** |
| **18** | **AT2G46810** | **MYC70** | **26** |  | **18** | **AT3G60030** | **SPL12** | **104** |
| **19** | **AT5G08330** | **CHE/TCP11^62^** | **26** |  | **19** | **AT5G15210** | **ZHD8** | **104** |
| **20** | **AT5G37260** | **RVE2^63^** | **26** |  | **20** | **AT1G50600** | **SCL5** | **103** |
| **21** | **AT5G44190** | **GLK2^64^** | **26** |  | **21** | **AT4G18890** | **BEH3^54^** | **103** |
| **22** | **AT1G58100** | **TCP8** | **25** |  | **22** | **AT5G61270** | **PIF7^60^** | **103** |
| **23** | **AT2G46830** | **CCA1^65^** | **25** |  | **23** | **AT2G18160** | **GBF5** | **102** |
| **24** | **AT4G39160** | **---** | **25** |  | **24** | **AT2G24650** | **---** | **102** |
| **25** | **AT5G57660** | **COL5^57^** | **25** |  | **25** | **AT3G10490** | **NAC052** | **102** |
| **26** | **AT5G61430** | **NAC100** | **25** |  | **26** | **AT5G12850** | **TZF8** | **102** |
| **27** | **AT1G02580** | **SDG5** | **24** |  | **27** | **AT3G04930** | **---** | **100** |
| **28** | **AT1G49720** | **ABF1** | **24** |  | **28** | **AT3G12980** | **HAC5** | **100** |
| **29** | **AT2G32700** | **MUM1** | **24** |  | **29** | **AT1G27050** | **---** | **99** |
| **30** | **AT3G19510** | **HAT3.1** | **24** |  | **30** | **AT2G44940** | **ERF34** | **99** |
| **31** | **AT1G04990** | **---** | **23** |  | **31** | **AT4G17750** | **HSFA1A^66^** | **99** |
| **32** | **AT1G30460** | **CPSF30** | **23** |  | **32** | **AT5G47640** | **NF-YB2** | **99** |
| **33** | **AT2G21320** | **BBX18** | **23** |  | **33** | **AT1G72740** | **---** | **98** |
| **34** | **AT4G35580** | **NTL9** | **23** |  | **34** | **AT1G76110** | **---** | **98** |
| **35** | **AT5G39860** | **PRE1** | **23** |  | **35** | **AT2G30590** | **WRKY21** | **98** |
| **36** | **AT5G59570** | **BOA** | **23** |  | **36** | **AT2G34450** | **---** | **98** |
| **37** | **AT1G74250** | **---** | **22** |  | **37** | **AT4G32570** | **TIFY8** | **98** |
| **38** | **AT3G14740** | **---** | **22** |  | **38** | **AT5G14140** | **---** | **98** |
| **39** | **AT4G00180** | **YAB3** | **22** |  | **39** | **AT5G45260** | **SLH1** | **98** |
| **40** | **AT4G05170** | **---** | **22** |  | **40** | **AT1G08000** | **GATA10** | **97** |
| **41** | **AT5G23420** | **HMGB6** | **22** |  | **41** | **AT1G63490** | **JMJ17** | **97** |
| **42** | **AT5G24470** | **PRR5^67^** | **22** |  | **42** | **AT2G23340** | **DEAR3** | **97** |
| **43** | **AT5G60100** | **PRR3^68^** | **22** |  | **43** | **AT2G33550** | **ASR3** | **97** |
| **44** | **AT1G20980** | **SPL1R2** | **21** |  | **44** | **AT3G22780** | **TSO1** | **97** |
| **45** | **AT3G07650** | **COL9** | **21** |  | **45** | **AT3G51950** | **---** | **97** |
| **46** | **AT4G22070** | **WRKY31** | **21** |  | **46** | **AT4G00990** | **JMJ27** | **97** |
| **47** | **AT4G23860** | **---** | **21** |  | **47** | **AT4G39780** | **---** | **97** |
| **48** | **AT5G29000** | **PHL1** | **21** |  | **48** | **AT1G32730** | **---** | **96** |
| **49** | **AT5G63280** | **---** | **21** |  | **49** | **AT2G22540** | **SVP** | **96** |
| **50** | **AT1G64860** | **SIG1^69^** | **20** |  | **50** | **AT2G43010** | **PIF4^55^** | **96** |

*The TF marked with red color on the yellow background is a known regulatory factor of this pathway. Frequency represents the number of genes regulated by each TF in the pathway.

**Table S9.** Regulatory genes identified and ranked by TGMI and SPLS for Light reaction pathway in *Arabidopsis thaliana*. Only the top 50 transcription factors that have the highest connectivity to pathway genes are shown.

| **TGMI** | | | |  | **SPLS** | | | |
| --- | --- | --- | --- | --- | --- | --- | --- | --- |
| **Rank** | **ID** | **Symbol** | **Freq.** |  | **Rank** | **ID** | **Symbol** | **Freq.** |
| **1** | **AT1G06040** | **STO/BBX24^53^** | **37** |  | **1** | **AT1G30970** | **SUF4** | **82** |
| **2** | **AT3G16350** | **---** | **35** |  | **2** | **AT4G23860** | **---** | **79** |
| **3** | **AT3G47500** | **CDF3** | **34** |  | **3** | **AT2G24650** | **---** | **77** |
| **4** | **AT5G15160** | **BNQ2^57^** | **31** |  | **4** | **AT4G35580** | **NTL9** | **77** |
| **5** | **AT2G45190** | **YAB1** | **30** |  | **5** | **AT5G61270** | **PIF7^60^** | **77** |
| **6** | **AT5G61270** | **PIF7^60^** | **30** |  | **6** | **AT1G19220** | **IAA22** | **76** |
| **7** | **AT3G59060** | **PIF5^55^** | **29** |  | **7** | **AT5G16560** | **KAN1** | **76** |
| **8** | **AT4G30860** | **SDG4** | **29** |  | **8** | **AT5G57660** | **COL5^57^** | **76** |
| **9** | **AT1G73870** | **COL7^56^** | **28** |  | **9** | **AT1G55970** | **HAG4** | **75** |
| **10** | **AT5G65210** | **TGA1** | **28** |  | **10** | **AT1G08010** | **GATA11** | **72** |
| **11** | **AT2G43010** | **PIF4^55^** | **27** |  | **11** | **AT3G23050** | **IAA7** | **71** |
| **12** | **AT5G37260** | **RVE2^63^** | **27** |  | **12** | **AT1G74250** | **---** | **70** |
| **13** | **AT3G60110** | **---** | **26** |  | **13** | **AT3G61830** | **ARF18** | **70** |
| **14** | **AT4G39160** | **---** | **26** |  | **14** | **AT4G26150** | **GNL^64^** | **70** |
| **15** | **AT5G08330** | **CHE/TCP11^62^** | **26** |  | **15** | **AT1G07530** | **SCL14** | **69** |
| **16** | **AT1G58100** | **TCP8** | **25** |  | **16** | **AT2G20280** | **---** | **69** |
| **17** | **AT5G57660** | **COL5^57^** | **25** |  | **17** | **AT2G22670** | **IAA8** | **69** |
| **18** | **AT5G61430** | **NAC100** | **25** |  | **18** | **AT1G58100** | **TCP8** | **67** |
| **19** | **AT2G31380** | **STH/BBX25^61^** | **24** |  | **19** | **AT1G69780** | **ATHB13** | **67** |
| **20** | **AT2G32700** | **MUM1** | **24** |  | **20** | **AT5G44190** | **GLK2^64^** | **67** |
| **21** | **AT3G19510** | **HAT3.1** | **24** |  | **21** | **AT1G70700** | **TIFY7** | **66** |
| **22** | **AT5G48250** | **---** | **24** |  | **22** | **AT1G06850** | **bZIP52** | **65** |
| **23** | **AT1G02580** | **SDG5** | **23** |  | **23** | **AT1G65620** | **AS2** | **65** |
| **24** | **AT1G34180** | **NAC016^59^** | **23** |  | **24** | **AT1G72740** | **---** | **65** |
| **25** | **AT1G74250** | **---** | **23** |  | **25** | **AT2G23340** | **DEAR3** | **65** |
| **26** | **AT4G05170** | **---** | **23** |  | **26** | **AT4G37790** | **HAT22** | **65** |
| **27** | **AT4G35580** | **NTL9** | **23** |  | **27** | **AT5G28300** | **---** | **65** |
| **28** | **AT5G39860** | **PRE1** | **23** |  | **28** | **AT1G20640** | **---** | **64** |
| **29** | **AT5G59570** | **BOA** | **23** |  | **29** | **AT1G53170** | **ERF8** | **64** |
| **30** | **AT3G14740** | **---** | **22** |  | **30** | **AT1G73870** | **COL7^56^** | **64** |
| **31** | **AT5G23420** | **HMGB6** | **22** |  | **31** | **AT2G41940** | **ZFP8** | **64** |
| **32** | **AT5G44190** | **GLK2^64^** | **22** |  | **32** | **AT3G51080** | **GATA6** | **64** |
| **33** | **AT5G67030** | **ZEP** | **22** |  | **33** | **AT1G06040** | **STO/BBX24^53^** | **63** |
| **34** | **AT1G20980** | **SPL1R2** | **21** |  | **34** | **AT1G13960** | **WRKY4** | **63** |
| **35** | **AT1G47870** | **E2FC** | **21** |  | **35** | **AT2G45190** | **YAB1** | **63** |
| **36** | **AT3G07650** | **COL9** | **21** |  | **36** | **AT5G26210** | **AL4** | **62** |
| **37** | **AT4G23860** | **---** | **21** |  | **37** | **AT2G22540** | **SVP** | **61** |
| **38** | **AT5G29000** | **PHL1** | **21** |  | **38** | **AT2G31230** | **ERF15** | **61** |
| **39** | **AT1G11950** | **---** | **20** |  | **39** | **AT3G28910** | **MYB30** | **61** |
| **40** | **AT2G21320** | **---** | **20** |  | **40** | **AT5G53200** | **TRY** | **61** |
| **41** | **AT5G13960** | **SUVH4** | **20** |  | **41** | **AT5G59780** | **MYB59** | **61** |
| **42** | **AT5G63280** | **---** | **20** |  | **42** | **AT5G61430** | **NAC100** | **61** |
| **43** | **AT5G15840** | **FG** | **19** |  | **43** | **AT4G00180** | **YAB3** | **60** |
| **44** | **AT5G16680** | **---** | **19** |  | **44** | **AT4G28790** | **---** | **60** |
| **45** | **AT1G18330** | **RVE7^70^** | **18** |  | **45** | **AT5G15160** | **BNQ2^57^** | **60** |
| **46** | **AT2G20280** | **---** | **18** |  | **46** | **AT1G46264** | **SCZ** | **59** |
| **47** | **AT2G46810** | **---** | **18** |  | **47** | **AT2G21530** | **---** | **59** |
| **48** | **AT4G31420** | **---** | **18** |  | **48** | **AT2G30424** | **TCL2** | **59** |
| **49** | **AT1G04250** | **IAA17** | **17** |  | **49** | **AT2G32700** | **MUM1** | **59** |
| **50** | **AT2G46830** | **CCA1^65^** | **17** |  | **50** | **AT3G19510** | **HAT3.1** | **59** |

*The TF marked with red color on the yellow background is a known regulatory factor of this pathway. Frequency represents the number of genes regulated by each TF in the pathway.

**References**

1 Saito, K. *et al.* The flavonoid biosynthetic pathway in Arabidopsis: structural and genetic diversity. *Plant physiology and biochemistry : PPB* **72**, 21-34, doi:10.1016/j.plaphy.2013.02.001 (2013).

2 Huang, J. *et al.* Functional analysis of the Arabidopsis PAL gene family in plant growth, development, and response to environmental stress. *Plant physiology* **153**, 1526-1538, doi:10.1104/pp.110.157370 (2010).

3 Wang, J. P. *et al.* Improving wood properties for wood utilization through multi-omics integration in lignin biosynthesis. *Nature communications* **9**, 1579, doi:10.1038/s41467-018-03863-z (2018).

4 Ehlting, J. *et al.* Three 4-coumarate:coenzyme A ligases in Arabidopsis thaliana represent two evolutionarily divergent classes in angiosperms. *The Plant journal : for cell and molecular biology* **19** (1999).

5 Kusano, M. *et al.* Metabolomics reveals comprehensive reprogramming involving two independent metabolic responses of Arabidopsis to UV-B light. *The Plant journal : for cell and molecular biology* **67**, 354-369, doi:10.1111/j.1365-313X.2011.04599.x (2011).

6 Appelhagen, I. *et al.* Update on transparent testa mutants from Arabidopsis thaliana: characterisation of new alleles from an isogenic collection. *Planta* **240**, 955-970, doi:10.1007/s00425-014-2088-0 (2014).

7 Kanehisa, M. & Goto, S. KEGG: kyoto encyclopedia of genes and genomes. *Nucleic Acids Res* **28**, 27-30 (2000).

8 Kanehisa, M., Sato, Y., Furumichi, M., Morishima, K. & Tanabe, M. New approach for understanding genome variations in KEGG. *Nucleic Acids Res* **47**, D590-D595, doi:10.1093/nar/gky962 (2019).

9 Kanehisa, M. Toward understanding the origin and evolution of cellular organisms. *Protein Sci* **28**, 1947-1951, doi:10.1002/pro.3715 (2019).

10 Tian, L., Xu, P., Chukhutsina, V. U., Holzwarth, A. R. & Croce, R. Zeaxanthin-dependent nonphotochemical quenching does not occur in photosystem I in the higher plant. *Proceedings of the National Academy of Sciences of the United States of America* **114**, 4828-4832, doi:10.1073/pnas.1621051114 (2017).

11 Nath, K. *et al.* Age-dependent changes in the functions and compositions of photosynthetic complexes in the thylakoid membranes of Arabidopsis thaliana. *Photosynthesis research* **117**, 547-556, doi:10.1007/s11120-013-9906-2 (2013).

12 Pan, X. *et al.* Structure of the maize photosystem I supercomplex with light-harvesting complexes I and II. *Science (New York, N.Y.)* **360**, 1109-1113, doi:10.1126/science.aat1156 (2018).

13 Jia, T., Ito, H. & Tanaka, A. Simultaneous regulation of antenna size and photosystem I/II stoichiometry in Arabidopsis thaliana. *Planta* **244**, 1041-1053 (2016).

14 Caspy, I. & Nelson, N. Structure of the plant photosystem I. *Biochemical Society transactions* **46**, 285-294, doi:10.1042/BST20170299 (2018).

15 Li, X.-P. *et al.* Regulation of photosynthetic light harvesting involves intrathylakoid lumen pH sensing by the PsbS protein. *The Journal of biological chemistry* **279**, 22866-22874 (2004).

16 Peterson, R. B. & Schultes, N. P. Light-harvesting complex B7 shifts the irradiance response of photosynthetic light-harvesting regulation in leaves of Arabidopsis thaliana. *Journal of plant physiology* **171**, 311-318, doi:10.1016/j.jplph.2013.09.007 (2014).

17 Elena López-Calcagno, P., Omar Abuzaid, A., Lawson, T. & Anne Raines, C. Arabidopsis CP12 mutants have reduced levels of phosphoribulokinase and impaired function of the Calvin-Benson cycle. *Journal of experimental botany* **68**, 2285-2298, doi:10.1093/jxb/erx084 (2017).

18 Gurrieri, L. *et al.* and phosphoribulokinase crystal structures complete the redox structural proteome of the Calvin-Benson cycle. *Proceedings of the National Academy of Sciences of the United States of America* **116**, 8048-8053, doi:10.1073/pnas.1820639116 (2019).

19 Li, W.-X. *et al.* The Arabidopsis NFYA5 transcription factor is regulated transcriptionally and posttranscriptionally to promote drought resistance. *The Plant cell* **20**, 2238-2251, doi:10.1105/tpc.108.059444 (2008).

20 Bechtold, U. *et al.* Time-Series Transcriptomics Reveals That AGAMOUS-LIKE22 Affects Primary Metabolism and Developmental Processes in Drought-Stressed Arabidopsis. *Plant Cell* **28**, 345-366, doi:10.1105/tpc.15.00910 (2016).

21 Mu, J., Tan, H., Hong, S., Liang, Y. & Zuo, J. Arabidopsis transcription factor genes NF-YA1, 5, 6, and 9 play redundant roles in male gametogenesis, embryogenesis, and seed development. *Molecular plant* **6**, 188-201, doi:10.1093/mp/sss061 (2013).

22 Xu, W., Dubos, C. & Lepiniec, L. Transcriptional control of flavonoid biosynthesis by MYB-bHLH-WDR complexes. *Trends in plant science* **20**, 176-185, doi:10.1016/j.tplants.2014.12.001 (2015).

23 O'Malley, R. C. *et al.* Cistrome and Epicistrome Features Shape the Regulatory DNA Landscape. *Cell* **165**, 1280-1292, doi:10.1016/j.cell.2016.04.038 (2016).

24 Kunieda, T. *et al.* NAC family proteins NARS1/NAC2 and NARS2/NAM in the outer integument regulate embryogenesis in Arabidopsis. *The Plant cell* **20**, 2631-2642, doi:10.1105/tpc.108.060160 (2008).

25 Lotkowska, M. E. *et al.* The Arabidopsis Transcription Factor MYB112 Promotes Anthocyanin Formation during Salinity and under High Light Stress. *Plant physiology* **169**, 1862-1880, doi:10.1104/pp.15.00605 (2015).

26 Datta, S., Hettiarachchi, C., Johansson, H. & Holm, M. SALT TOLERANCE HOMOLOG2, a B-box protein in Arabidopsis that activates transcription and positively regulates light-mediated development. *The Plant cell* **19**, 3242-3255 (2007).

27 Nakabayashi, R. *et al.* Enhancement of oxidative and drought tolerance in Arabidopsis by overaccumulation of antioxidant flavonoids. *The Plant journal : for cell and molecular biology* **77**, 367-379, doi:10.1111/tpj.12388 (2014).

28 Wang, X.-C. *et al.* Arabidopsis MYB4 plays dual roles in flavonoid biosynthesis. *The Plant journal : for cell and molecular biology* **101**, 637-652, doi:10.1111/tpj.14570 (2020).

29 Zhang, Y., Zheng, S., Liu, Z., Wang, L. & Bi, Y. Both HY5 and HYH are necessary regulators for low temperature-induced anthocyanin accumulation in Arabidopsis seedlings. *Journal of plant physiology* **168**, 367-374, doi:10.1016/j.jplph.2010.07.025 (2011).

30 Wang, J. *et al.* Overexpression of BoNAC019, a NAC transcription factor from Brassica oleracea, negatively regulates the dehydration response and anthocyanin biosynthesis in Arabidopsis. *Scientific reports* **8**, 13349, doi:10.1038/s41598-018-31690-1 (2018).

31 Gupta, O. P. *et al.* Conserved miRNAs modulate the expression of potential transcription factors of isoflavonoid biosynthetic pathway in soybean seeds. *Molecular biology reports* **46**, 3713-3730, doi:10.1007/s11033-019-04814-7 (2019).

32 Geng, P. *et al.* MYB20, MYB42, MYB43, and MYB85 Regulate Phenylalanine and Lignin Biosynthesis during Secondary Cell Wall Formation. *Plant physiology* **182**, 1272-1283, doi:10.1104/pp.19.01070 (2020).

33 Zhong, R. & Ye, Z.-H. The Arabidopsis NAC transcription factor NST2 functions together with SND1 and NST1 to regulate secondary wall biosynthesis in fibers of inflorescence stems. *Plant signaling & behavior* **10**, e989746, doi:10.4161/15592324.2014.989746 (2015).

34 Zhou, J., Lee, C., Zhong, R. & Ye, Z.-H. MYB58 and MYB63 are transcriptional activators of the lignin biosynthetic pathway during secondary cell wall formation in Arabidopsis. *The Plant cell* **21**, 248-266, doi:10.1105/tpc.108.063321 (2009).

35 Zhong, R., Lee, C. & Ye, Z.-H. Global analysis of direct targets of secondary wall NAC master switches in Arabidopsis. *Molecular plant* **3**, 1087-1103, doi:10.1093/mp/ssq062 (2010).

36 Taylor-Teeples, M. *et al.* An Arabidopsis gene regulatory network for secondary cell wall synthesis. *Nature* **517**, 571-575, doi:10.1038/nature14099 (2015).

37 Ohashi-Ito, K., Iwamoto, K. & Fukuda, H. LOB DOMAIN-CONTAINING PROTEIN 15 Positively Regulates Expression of VND7, a Master Regulator of Tracheary Elements. *Plant & cell physiology* **59**, 989-996, doi:10.1093/pcp/pcy036 (2018).

38 Liu, C., Yu, H. & Li, L. SUMO modification of LBD30 by SIZ1 regulates secondary cell wall formation in Arabidopsis thaliana. *PLoS genetics* **15**, e1007928, doi:10.1371/journal.pgen.1007928 (2019).

39 Öhman, D. *et al.* MYB103 is required for FERULATE-5-HYDROXYLASE expression and syringyl lignin biosynthesis in Arabidopsis stems. *The Plant journal : for cell and molecular biology* **73**, 63-76, doi:10.1111/tpj.12018 (2013).

40 Zhong, R. & Ye, Z.-H. MYB46 and MYB83 bind to the SMRE sites and directly activate a suite of transcription factors and secondary wall biosynthetic genes. *Plant & cell physiology* **53**, 368-380, doi:10.1093/pcp/pcr185 (2012).

41 Zhong, R., Lee, C., Zhou, J., McCarthy, R. L. & Ye, Z.-H. A battery of transcription factors involved in the regulation of secondary cell wall biosynthesis in Arabidopsis. *The Plant cell* **20**, 2763-2782, doi:10.1105/tpc.108.061325 (2008).

42 Agarwal, T., Grotewold, E., Doseff, A. I. & Gray, J. MYB31/MYB42 Syntelogs Exhibit Divergent Regulation of Phenylpropanoid Genes in Maize, Sorghum and Rice. *Scientific reports* **6**, 28502, doi:10.1038/srep28502 (2016).

43 Chai, G. *et al.* Arabidopsis C3H14 and C3H15 have overlapping roles in the regulation of secondary wall thickening and anther development. *Journal of experimental botany* **66**, 2595-2609, doi:10.1093/jxb/erv060 (2015).

44 Wang, H. *et al.* Mutation of WRKY transcription factors initiates pith secondary wall formation and increases stem biomass in dicotyledonous plants. *Proceedings of the National Academy of Sciences of the United States of America* **107**, 22338-22343, doi:10.1073/pnas.1016436107 (2010).

45 Zhou, J., Zhong, R. & Ye, Z.-H. Arabidopsis NAC domain proteins, VND1 to VND5, are transcriptional regulators of secondary wall biosynthesis in vessels. *PloS one* **9**, e105726, doi:10.1371/journal.pone.0105726 (2014).

46 Liu, Y. *et al.* BEL1-LIKE HOMEODOMAIN6 and KNOTTED ARABIDOPSIS THALIANA7 interact and regulate secondary cell wall formation via repression of REVOLUTA. *The Plant cell* **26**, 4843-4861, doi:10.1105/tpc.114.128322 (2014).

47 Zhao, C. *et al.* XYLEM NAC DOMAIN1, an angiosperm NAC transcription factor, inhibits xylem differentiation through conserved motifs that interact with RETINOBLASTOMA-RELATED. *The New phytologist* **216**, 76-89, doi:10.1111/nph.14704 (2017).

48 Nishitani, K. & Demura, T. Editorial: an emerging view of plant cell walls as an apoplastic intelligent system. *Plant & cell physiology* **56**, 177-179, doi:10.1093/pcp/pcv001 (2015).

49 Soyano, T., Thitamadee, S., Machida, Y. & Chua, N.-H. ASYMMETRIC LEAVES2-LIKE19/LATERAL ORGAN BOUNDARIES DOMAIN30 and ASL20/LBD18 regulate tracheary element differentiation in Arabidopsis. *The Plant cell* **20**, 3359-3373, doi:10.1105/tpc.108.061796 (2008).

50 Zhang, J. *et al.* Transcriptional regulatory framework for vascular cambium development in Arabidopsis roots. *Nature plants* **5**, 1033-1042, doi:10.1038/s41477-019-0522-9 (2019).

51 Oh, S., Park, S. & Han, K. H. Transcriptional regulation of secondary growth in Arabidopsis thaliana. *J Exp Bot* **54**, 2709-2722, doi:10.1093/jxb/erg304 (2003).

52 Endo, H. *et al.* Multiple classes of transcription factors regulate the expression of VASCULAR-RELATED NAC-DOMAIN7, a master switch of xylem vessel differentiation. *Plant & cell physiology* **56**, 242-254, doi:10.1093/pcp/pcu134 (2015).

53 Job, N., Yadukrishnan, P., Bursch, K., Datta, S. & Johansson, H. Two B-Box Proteins Regulate Photomorphogenesis by Oppositely Modulating HY5 through their Diverse C-Terminal Domains. *Plant physiology* **176**, 2963-2976, doi:10.1104/pp.17.00856 (2018).

54 Oh, E., Zhu, J.-Y. & Wang, Z.-Y. Interaction between BZR1 and PIF4 integrates brassinosteroid and environmental responses. *Nature cell biology* **14**, 802-809, doi:10.1038/ncb2545 (2012).

55 Kim, K. *et al.* PIF1 Regulates Plastid Development by Repressing Photosynthetic Genes in the Endodermis. *Molecular plant* **9**, 1415-1427, doi:10.1016/j.molp.2016.08.007 (2016).

56 Zhang, Z. *et al.* CONSTANS-LIKE 7 (COL7) is involved in phytochrome B (phyB)-mediated light-quality regulation of auxin homeostasis. *Molecular plant* **7**, 1429-1440, doi:10.1093/mp/ssu058 (2014).

57 Richter, R., Behringer, C., Müller, I. K. & Schwechheimer, C. The GATA-type transcription factors GNC and GNL/CGA1 repress gibberellin signaling downstream from DELLA proteins and PHYTOCHROME-INTERACTING FACTORS. *Genes & development* **24**, 2093-2104, doi:10.1101/gad.594910 (2010).

58 Liu, X. *et al.* EIN3 and PIF3 Form an Interdependent Module That Represses Chloroplast Development in Buried Seedlings. *The Plant cell* **29**, 3051-3067, doi:10.1105/tpc.17.00508 (2017).

59 Sakuraba, Y., Han, S.-H., Lee, S.-H., Hörtensteiner, S. & Paek, N.-C. Arabidopsis NAC016 promotes chlorophyll breakdown by directly upregulating STAYGREEN1 transcription. *Plant cell reports* **35**, 155-166, doi:10.1007/s00299-015-1876-8 (2016).

60 Jiang, Y. *et al.* The ELF3-PIF7 Interaction Mediates the Circadian Gating of the Shade Response in Arabidopsis. *iScience* **22**, 288-298, doi:10.1016/j.isci.2019.11.029 (2019).

61 Gangappa, S. N. *et al.* The Arabidopsis B-BOX protein BBX25 interacts with HY5, negatively regulating BBX22 expression to suppress seedling photomorphogenesis. *The Plant cell* **25**, 1243-1257, doi:10.1105/tpc.113.109751 (2013).

62 Pruneda-Paz, J. L., Breton, G., Para, A. & Kay, S. A. A functional genomics approach reveals CHE as a component of the Arabidopsis circadian clock. *Science (New York, N.Y.)* **323**, 1481-1485, doi:10.1126/science.1167206 (2009).

63 Zhang, X. *et al.* Constitutive expression of CIR1 (RVE2) affects several circadian-regulated processes and seed germination in Arabidopsis. *The Plant journal : for cell and molecular biology* **51**, 512-525 (2007).

64 Zubo, Y. O. *et al.* Coordination of Chloroplast Development through the Action of the GNC and GLK Transcription Factor Families. *Plant physiology* **178**, 130-147, doi:10.1104/pp.18.00414 (2018).

65 Andronis, C., Barak, S., Knowles, S. M., Sugano, S. & Tobin, E. M. The clock protein CCA1 and the bZIP transcription factor HY5 physically interact to regulate gene expression in Arabidopsis. *Molecular plant* **1**, 58-67, doi:10.1093/mp/ssm005 (2008).

66 Yoshida, T. *et al.* Arabidopsis HsfA1 transcription factors function as the main positive regulators in heat shock-responsive gene expression. *Molecular genetics and genomics : MGG* **286**, 321-332, doi:10.1007/s00438-011-0647-7 (2011).

67 Nakamichi, N. *et al.* PSEUDO-RESPONSE REGULATORS 9, 7, and 5 are transcriptional repressors in the Arabidopsis circadian clock. *The Plant cell* **22**, 594-605, doi:10.1105/tpc.109.072892 (2010).

68 Nakamichi, N., Kita, M., Ito, S., Yamashino, T. & Mizuno, T. PSEUDO-RESPONSE REGULATORS, PRR9, PRR7 and PRR5, together play essential roles close to the circadian clock of Arabidopsis thaliana. *Plant & cell physiology* **46**, 686-698 (2005).

69 Shimizu, M. *et al.* Sigma factor phosphorylation in the photosynthetic control of photosystem stoichiometry. *Proceedings of the National Academy of Sciences of the United States of America* **107**, 10760-10764, doi:10.1073/pnas.0911692107 (2010).

70 Kuno, N. *et al.* The novel MYB protein EARLY-PHYTOCHROME-RESPONSIVE1 is a component of a slave circadian oscillator in Arabidopsis. *The Plant cell* **15**, 2476-2488 (2003).
